# The Early Development of the Blue‐Eye Cichlid, *Cryptoheros spilurus* (Cichliformes: Cichlidae)

**DOI:** 10.1111/ede.70019

**Published:** 2025-10-04

**Authors:** Rubén Alonso Contreras‐Tapia, Jairo Arroyave, Gabriela Garza‐Mouriño, María Elena Castellanos‐Páez, Marcela Ivonne Benítez‐Díaz Mirón, Wilfredo A. Matamoros

**Affiliations:** ^1^ Laboratorio de Rotiferología y Biología Molecular de Plancton, Departamento El Hombre y su Ambiente Universidad Autónoma Metropolitana, Unidad Xochimilco (UAM‐X) Ciudad de México México; ^2^ Instituto de Biología Universidad Nacional Autónoma de México (UNAM) Ciudad de México México; ^3^ Laboratorio de Diversidad Acuática y Biogeografía, Instituto de Ciencias Biológicas Universidad de Ciencias y Artes de Chiapas (UNICACH), Tuxtla Gutiérrez Chiapas México; ^4^ Instituto Hondureño de Investigación en Ciencias Biológicas y Ambientales de Honduras (IBIOAH), Facultad de Ciencias Universidad Nacional Autónoma de Honduras (UNAH), Campus Cortés San Pedro Sula Honduras; ^5^ Collections Associate Field Museum of Natural History Chicago Illinois USA

**Keywords:** larval development, morphometry, neotropical, ultrastructure

## Abstract

The early development of *Cryptoheros spilurus*, a substrate‐breeding Middle American cichlid, was studied from hatching to 16 days post‐hatching (dph), to document for the first time, the sequence of key ontogenetic changes. Eggs, deposited on rocky substrates, measured 1.65 ± 0.05 mm in diameter, with asynchronous hatching occurring at 52–54 h post‐fertilization. Hatchlings (TL = 4.739 ± 0.27 mm) showed a large yolk sacs, finfold, straight notochord, and undeveloped eyes. Scanning electron microscopy revealed early differentiation of structures, including cement glands, olfactory pits, and optic primordia. Cement glands, previously reported in other cichlids, were documented here in their full developmental chronology, including their regression by 7 dph. Cranial development proceeded rapidly, with pigmentation and eye formation initiating by 1 dph and oral cavity, dentition, and taste buds forming by 6 dph. Fin development followed a sequential pattern: early pectoral fin formation supported initial mobility, while caudal, dorsal, anal, and pelvic fins emerged progressively, with full formation completed by 16 dph. Pigmentation evolved from a ventral melanophore stripe to a distinct species‐specific pattern involving xanthophores and iridophores. By 16 dph, *C. spilurus* had completed metamorphosis (TL = 13.168 ± 0.55 mm). Allometric analysis revealed biphasic growth trajectories. Structures involved in feeding and sensory input, such as head length, snout length, and gape size, exhibited prolonged positive allometry, while trunk and tail traits showed delayed or negative allometry. These patterns reflect functional prioritization during the shift to active foraging. This study highlights *C. spilurus* as a valuable model for examining heterochrony, morphological modularity, and ecological adaptation during early development. Our findings provide essential baseline data for future comparative work on developmental plasticity and diversification in Neotropical cichlids.

## Summary

- Early development of Cryptoheros spilurus was described from hatching to 16 days post‐hatching using SEM and morphometrics.
- Key structures such as cement glands, taste buds, and fins formed rapidly.
- Distinct pigment patterns and allometric growth trajectories mark metamorphosis at 16 dph.

## Graphical abstract

We document the early development of *Cryptoheros spilurus*, highlighting rapid cranial differentiation, sensory organ formation, and fin development. Ontogenetic allometry and ultrastructural traits reveal functional adaptations for early foraging and locomotion, offering insights into ecological success and the developmental basis of cichlid diversification.

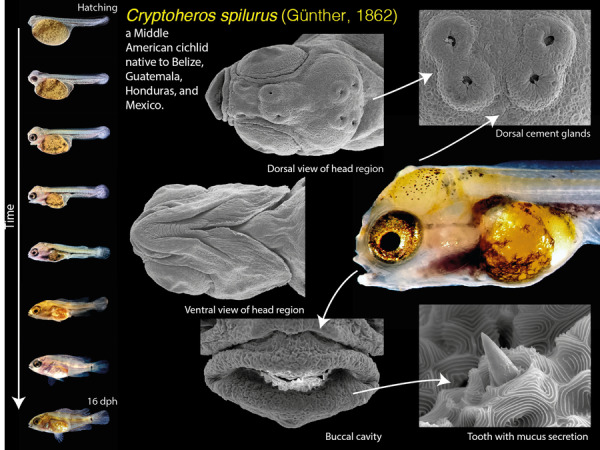

## 1 Introduction

Fishes exhibit remarkable diversity in their developmental strategies, reflecting adaptations to ecological conditions, reproductive investment, and their evolutionary history. Broadly, fish development can be classified into two major modes: direct and indirect development (Flegler‐Balon 1989; Balon 1999; Osse et al. 1997; Miller and Kendall 2009). In direct development, offspring hatch or are born in an advanced state, resembling miniature versions of the adult. These juveniles typically exhibit functional mouths, pigmented eyes, and differentiated fins at hatching, and often begin exogenous feeding immediately. This strategy is commonly associated with large, yolk‐rich eggs, prolonged embryogenesis, and increased parental care (Flegler‐Balon 1989; Miller and Kendall 2009; Schneider et al. 2023). Many freshwater fishes, particularly those that lay demersal eggs or exhibit brooding behaviors, follow this strategy. For example, African mouthbrooding cichlids such as *Astatotilapia* spp. and *Oreochromis niloticus* produce well‐developed offspring that emerge with advanced skeletal structures, bypassing a distinct larval stage (Fujimura and Okada 2007; Woltering et al. 2018; Marconi et al. 2023). Similarly, some syngnathids (e.g., *Syngnathus typhle, Hippocampus erectus*) give birth to juvenile‐like offspring following internal brooding (Schneider et al. 2023). In contrast, indirect development involves a distinct larval stage with transient structures and a morphologically simplified body plan. Hatchlings emerge in a relatively undeveloped state, often as yolk‐sac larvae, with underdeveloped sensory, locomotor, and digestive systems. These larvae rely on yolk reserves while critical structures develop, and only later transition to active feeding and swimming (Flegler‐Balon 1989; Balon 1999; Miller and Kendall 2009). Typically associated with numerous, small eggs, this strategy is widespread in both marine and freshwater taxa (e.g., *Pagrus major*, *Cichlasoma dimerus*, and *Nannacara anomala*; Osse and Van den Boogaart 1995; Kupren et al. 2014). A hallmark of indirect development is the presence of specialized larval structures, such as a median finfold, temporary head spines, or cement glands, which are reabsorbed or transformed during a metamorphic transition into the juvenile form.

Even within taxonomic groups, variation in developmental strategies is evident. Among cichlids, substrate‐brooding species such as *Cichlasoma dimerus* and *Amphilophus xiloaensis* exhibit traits consistent with indirect development, including poorly ossified hatchlings, persistent finfolds, and delayed differentiation of adult structures (Meijide and Guerrero 2000; Kratochwil et al. 2015; Beriotto et al. 2023). In these species, traits such as cement glands, respiratory plexuses, and a persistent median finfold are observed during early stages but regress as metamorphosis proceeds. Conversely, mouthbrooding cichlids often display direct development, with larvae emerging well‐formed and rapidly transitioning to juvenile morphology (Balon 1999; Fujimura and Okada 2007; Saemi‐Komsari et al. 2018). These contrasting developmental modes are not merely of taxonomic interest; they reflect deep evolutionary trade‐offs between offspring number and quality, timing of independence, and ecological niche exploitation (Balon 1999). Furthermore, they offer powerful systems for comparative evolutionary developmental biology. Teleost fishes, including zebrafish (*Danio rerio*) and medaka (*Oryzias latipes*), have long served as model organisms to study indirect development (Iwamatsu 2004; Parichy et al. 2009), yet many ecologically and evolutionarily important lineages, such as Neotropical cichlids, remain poorly characterized at early ontogenetic stages.

The larval stage in fishes represents a critical developmental period characterized by profound morphological and physiological transformations that occur from hatching until the fish becomes a juvenile (Rønnestad et al. 2013; Finn 2020). During this stage, temporary structures support essential functions required for early survival. For instance, the larval finfold, composed of major and minor lobes, facilitates locomotion and is gradually resorbed during ontogeny (Fujimura and Okada 2007; Parichy et al. 2009). Cement glands, which secrete adhesive mucus, enable substrate attachment immediately after hatching but are lost as larvae mature (Groppelli et al. 2003; Nelson et al. 2019; Contreras‐Tapia et al. 2024b). Similarly, the yolk sac provides endogenous nutrition and is gradually resorbed, marking the transition to exogenous feeding, a key developmental milestone often associated with a mixed feeding phase (Fujimura and Okada 2007; Yúfera and Darias 2007; Contreras‐Tapia et al. 2024a). These transient features support survival during early development but are remodeled or lost as the larva undergoes metamorphosis into the juvenile form.

Environmental factors, particularly temperature and oxygen availability, strongly influence developmental timing and outcomes (Kratochwil et al. 2015; Schneider et al. 2023). This environmental sensitivity enables developmental plasticity, allowing larvae to synchronize progression with optimal environmental conditions. In cichlids, such plasticity has been a key factor in their remarkable adaptive radiation, facilitating the exploitation of diverse ecological niches (Osse and Van den Boogaart 1995; Parichy et al. 2009; Marconi et al. 2023). For instance, indirect development can result in heterogeneous growth patterns during the onset of exogenous feeding. These variations reflect the plasticity of developmental pathways and their influence on morphological diversification, a fundamental process underpinning evolutionary adaptation in cichlids (Kratochwil et al. 2015; Hu and Albertson 2017; Marconi et al. 2023).

Developmental plasticity often manifests through heterochrony, changes in developmental processes' timing, rate, or duration. These shifts can give rise to morphological variation, particularly in structures such as cranial and trunk regions, which originate from early differences in somite formation (Albertson and Kocher 2006; Marconi et al. 2023). Studying these temporal shifts provides insights into the interplay of genetic, cellular, and environmental factors that shape phenotypic diversity and evolutionary trajectories. Cichlids are a well‐established model system for investigating how early developmental changes contribute to evolutionary diversification, particularly due to their remarkable ecological and morphological diversity (Albertson and Kocher 2006; Burress 2015; Hulsey et al. 2019; Marconi et al. 2023). By focusing on larval stages, researchers can explore the origins of adult morphology, ecological adaptation, and speciation (Albertson and Kocher 2006; Powder and Albertson 2016). This focus situates larval development at the intersection of evolutionary developmental biology (Evo‐Devo) and ecological adaptation, offering a framework for understanding the generation and maintenance of biodiversity.

Fishes such as zebrafish, medaka, and cichlids have become models for studying developmental processes, gene function, and environmental responsiveness (Albertson 2003; Albertson and Kocher 2006; Parichy et al. 2009; Powder and Albertson 2016; Marconi et al. 2023). Staging tables, which categorize embryonic and larval development based on morphological criteria, such as blastomere number, blastoderm morphology, epiboly progression, and organogenesis, enable standardized comparisons across species and experimental contexts (Fujimura and Okada 2007; Hopwood 2007). These developmental stages are also widely used in evolutionary and applied studies, from identifying the origins of morphological novelty (Woltering et al. 2018; Powder and Albertson 2016; Conith et al. 2019) to evaluating heterochrony in cranial or axial patterning (Powder et al. 2015; Marconi et al. 2023; Prazdnikov 2024). Beyond Evo‐Devo, larval stages are pivotal in ecotoxicology for assessing pollutant effects (Sfakianakis et al. 2015) and in aquaculture for diagnosing skeletal deformities and optimizing rearing conditions (Cahu et al. 2003; Boglione et al. 2013). The study of larval development in cichlids offers key insights into how form and function evolve in response to ecological pressures, positioning this group of fishes as an exciting and promising model in integrative biological research.

Commonly known as the blue‐eye cichlid, *Cryptoheros spilurus* (Günther, 1862) is a small Middle American cichlid widely distributed in freshwater environments across Belize, Guatemala, Mexico, and Honduras (Artigas‐Azas 2007; Říčan et al. 2016; Buege et al. 2021). This species has already been used in evolutionary developmental biology studies, particularly to investigate the roles of hormones and pigmentation in phenotypic variation (Prazdnikov and Shkil 2023; Prazdnikov 2024). In this study, we investigated the early development of *C. spilurus*, with a focus on larval morphological differentiation and morphometric trajectories. It inhabits a variety of habitats, including lakes with sandy substrates, slow‐moving creeks, and river pools. For instance, in Lake Caobas (Yucatán Peninsula, Mexico), it occurs in systems characterized by subterranean water circulation and surrounding vegetation such as subperennifolia forests and secondary growth (Valtierra‐Vega and Schmitter‐Soto 2000). In contrast, in the upper Bladen River (Belize), *C. spilurus* has been observed maneuvering through cobble substrates in swift currents, although it is less associated with sand substrates for nesting compared to other cichlids (Buege et al. 2021). These observations suggest a high degree of ecological flexibility, enabling the species to exploit a wide range of mesohabitats for feeding and reproduction. *Cryptoheros spilurus* belongs to the Heroini clade (Amphilophines), a speciose tribe of Neotropical cichlids that radiated extensively throughout Middle America (Říčan et al. 2016; Alda et al. 2021). Within Heroini, the genus *Cryptoheros* forms part of a monophyletic group of small‐bodied, substrate‐brooding cichlids whose phylogenetic relationships are increasingly well‐resolved but still present questions regarding trait evolution and ecological diversification. Developmental data from *C. spilurus* provide a valuable opportunity to investigate early life‐history traits in the context of the Neotropical cichlid lineage, allowing comparisons with related taxa to examine whether larval characters correlate with environmental specialization, parental care strategies, or miniaturization, key themes in cichlid evolution (Burress 2015; Kratochwil et al. 2015; Powder et al. 2015).

We adopt the term “larval stage” to describe the post‐hatching period marked by transient morphological features such as cement glands and a continuous median finfold, consistent with the definitions proposed by Flegler‐Balon (1989) and Balon (1999). While *C. spilurus* does not undergo a dramatic metamorphosis as in many marine teleosts, these temporary structures indicate a distinct post‐hatching developmental phase before juvenile differentiation. Moreover, *C. spilurus* possesses several traits that make it a promising candidate for use as a model cichlid species: substrate‐brooding species with biparental care, small body size, tolerance to varied environmental conditions, ease of maintenance in captivity, and well‐documented reproductive behavior. In contrast to the African cichlid models widely used in evo‐devo and behavioral studies (e.g., *Astatotilapia burtoni, Oreochromis niloticus*), Middle American cichlids remain underrepresented in developmental research, limiting our understanding of how key traits have evolved across the cichlid radiation.

Early ontogenetic data from *C. spilurus* can help bridge this gap and enable broader comparative studies across geographic lineages. This paper represents a first step toward understanding intraspecific variation in early developmental traits, with the broader goal of revealing intra‐ and interspecific patterns of geographic variability in these traits. By characterizing early ontogeny in this ecologically versatile species, we contribute to the foundational knowledge necessary to explore the evolution of development in Neotropical cichlids. While this study does not directly examine phenotypic plasticity in relation to ecological variation, the detailed descriptive data provided here form an essential baseline for future investigations. By providing the first detailed ontogenetic description of *C. spilurus*, our work lays the foundation for future hypothesis‐driven research on the evolution of larval morphology and phenotypic plasticity in Neotropical cichlids. Such baseline developmental data are essential for understanding how life‐history traits evolve in response to ecological pressures.

## 2 Materials and Methods

### 2.1 Breeding of *Cryptoheros spilurus*

Adult individuals of *Cryptoheros spilurus* were maintained under controlled laboratory conditions for a minimum of 2 years at the Laboratorio de Rotiferología y Biología Molecular de Plancton, Universidad Autónoma Metropolitana‐Xochimilco, Mexico (UAM‐X). Two replicate breeding groups, each comprising four adult males and four females, were housed in separate 240 L glass aquaria (120 × 40 × 50 cm). The tanks were equipped with flagstone rocks and clay pots to provide shelter and spawning substrates. Each aquarium was fitted with continuous aeration and mechanical, chemical, and biological filtration systems. A week replacement of 10% tank volume ensured consistent appropriate water quality. The following parameters were maintained: pH = 8.0 ± 0.5, temperature = 28 ± 1°C, dissolved oxygen = 6.0 ± 0.5 mg/L, and a 12:12‐h light/dark photoperiod. Fish were fed three times daily with a commercial omnivorous diet (El Pedregal; 32% protein content).

Under these conditions, females spawned at regular intervals of 15 to 18 days, producing clutches of 200 to 500 eggs. Oviposition typically occurred on the provided rocks or inside clay pots, which were subsequently transferred to 40 L aquaria filled with water from parental tank to ensure identical environmental conditions. To monitor embryonic development, 10 eggs were randomly selected from the clutch every 3 h and observed under a stereomicroscope. After each observation, the eggs were transferred to a separate 40 L aquarium and not returned to the clutch. At each time point, a new set of 10 eggs was sampled from the same clutch. This approach was used to minimize potential effects of repeated manipulation and exposure to stereomicroscopy on embryonic development. The observations were repeated until hatching (52–54 h post‐fertilization). Hatching was defined as the moment when the larva had completely exited the chorion. In *C. spilurus*, this process occurred rapidly, typically full emergence was within a few seconds. Post‐hatching, the larvae remained in the same tank for continued observation. Multiple clutches were followed using a standardized developmental protocol to characterize morphological changes, growth rates, and behavioral milestones until 16 days post‐hatch (dph).

### 2.2 Sampling and Imaging

A total of 30 randomly selected eggs and larvae were sampled to document developmental progression. During the first three dph, observations were conducted at 6‐h intervals; thereafter, observations occurred every 12 h. All observations were performed on live specimens under ambient conditions to ensure accurate documentation of morphological and functional characteristics. Developmental age was recorded in both hours post‐hatch (hph) and dph, with hatching designated as time zero. Morphological changes were initially documented with images and measurements using a Nikon SMZ1500 stereomicroscope equipped with a high‐resolution microscope digital camera (Olympus DP72). A Nikon NI‐30 fiber optic illuminator provided consistent illumination. These images were analyzed using Image‐Pro Plus v7.1 (Media Cybernetics, Silver Spring, MD, USA), calibrated with an Olympus 1‐mm stage micrometer.

In addition to documenting ontogenetic changes by means of optical stereo microscopy, from 0 to 7 dph, five larvae were randomly selected daily for imaging via scanning electron microscopy (SEM). For this, larvae were euthanized with tricaine methanesulfonate (MS‐222, 500 mg/L), then fixed in 4% formaldehyde in phosphate‐buffered saline (PBS; pH 7.4) for 12 h at 4°C. Samples were dehydrated in a graded ethanol series (10%, 30%, 50%, 70%, 90%, 100%; 3 h each), followed by critical point drying using CO_2_. Dried larvae were mounted on metal stubs using a carbon adhesive and sputter‐coated with gold. SEM imaging was conducted using a Hitachi Stereoscan SU1510 SEM at 10 kV at the Laboratorio de Microscopia y Fotografía de la Biodiversidad 1 (LaNaBio) of the Instituto de Biología, Universidad Nacional Autónoma de México. Images were then processed in Image‐Pro Plus v7.1.

### 2.3 Morphometric Data Collection and Analysis

Given the nonspherical morphology of the eggs, the principal axis (Y) and minor axis (X) were measured. The effective diameter (*d*_*e*_) was calculated using the formula: $d_{e}={\left(\frac{Y}{X^{2}}\right)}^{1/3}$ following the method of Coleman (1991). Measurements of yolk sac volume were excluded because its shape changed markedly over time, transitioning from ovoid to irregular during development, making accurate and consistent quantification impractical. Larval development was characterized using the following morphometric parameters: total length (TL), standard length (SL), head length (HL), trunk length (TRL), tail length (post‐anal; TAL), eye diameter (ED), and body depth (BD). TL was measured differently depending on the developmental stage: in pre‐oral opening larvae, TL was defined as the longest linear anteroposterior axis; in post‐oral opening larvae, it extended from the anterior tip of the upper jaw to the posterior edge of the caudal fin, following Fujimura and Okada (2007). Gape size (GS) was assessed as a proxy for feeding capability, using a modified equation adapted by GUMA'A (1978): $\mathrm{GS}=\;\sqrt{{\mathrm{UJL}}^{2}+{\mathrm{LJL}}^{2}}$; where UJL and LJL represent upper and lower jaw lengths, respectively. All measurements were expressed in millimeters as mean values ± standard deviation. Daily morphometrics were obtained from 15 larvae per day (*N* = 255), and GS was evaluated in a subset of 228 individuals.

Morphometric data were analyzed using the power function *y = ax* ^*b*^, where *y* is the dependent variable (measured character), *x* the independent variable (TL), *a* is the intercept, and *b* is the growth coefficient. Isometric growth was defined by *b* = 1; *b* > 1 indicated positive allometry and *b* < 1 indicated negative allometry. Student's *t*‐test was applied to test significant deviation of *b* from 1, using the formula: *t* = (*b*−*b*_*0*_)/*S*_*b*_, where *b* is the estimated exponent from the regression, *b*_*0*_ is the hypothesized value, and *S*_*b*_ is the standard error of *b*.

To identify developmental inflection points, the approach of Van Snik et al. (1997) was followed. The data set was divided into two segments at varying intermediate x‐values, and linear regressions were fitted to each subset. The x‐value associated with the greatest difference in slope (*t‐*value) between segments was defined as the inflection point. Developmental inflection points were identified as the values of total length (TL) at which the slope of allometric growth significantly changed, indicating shifts in functional growth priorities. These *t‐*values were calculated for identification only and were not reported in the results.

## 3 Results

### 3.1 Overview of Early Ontogeny and Developmental Milestones

**Figure 1:**
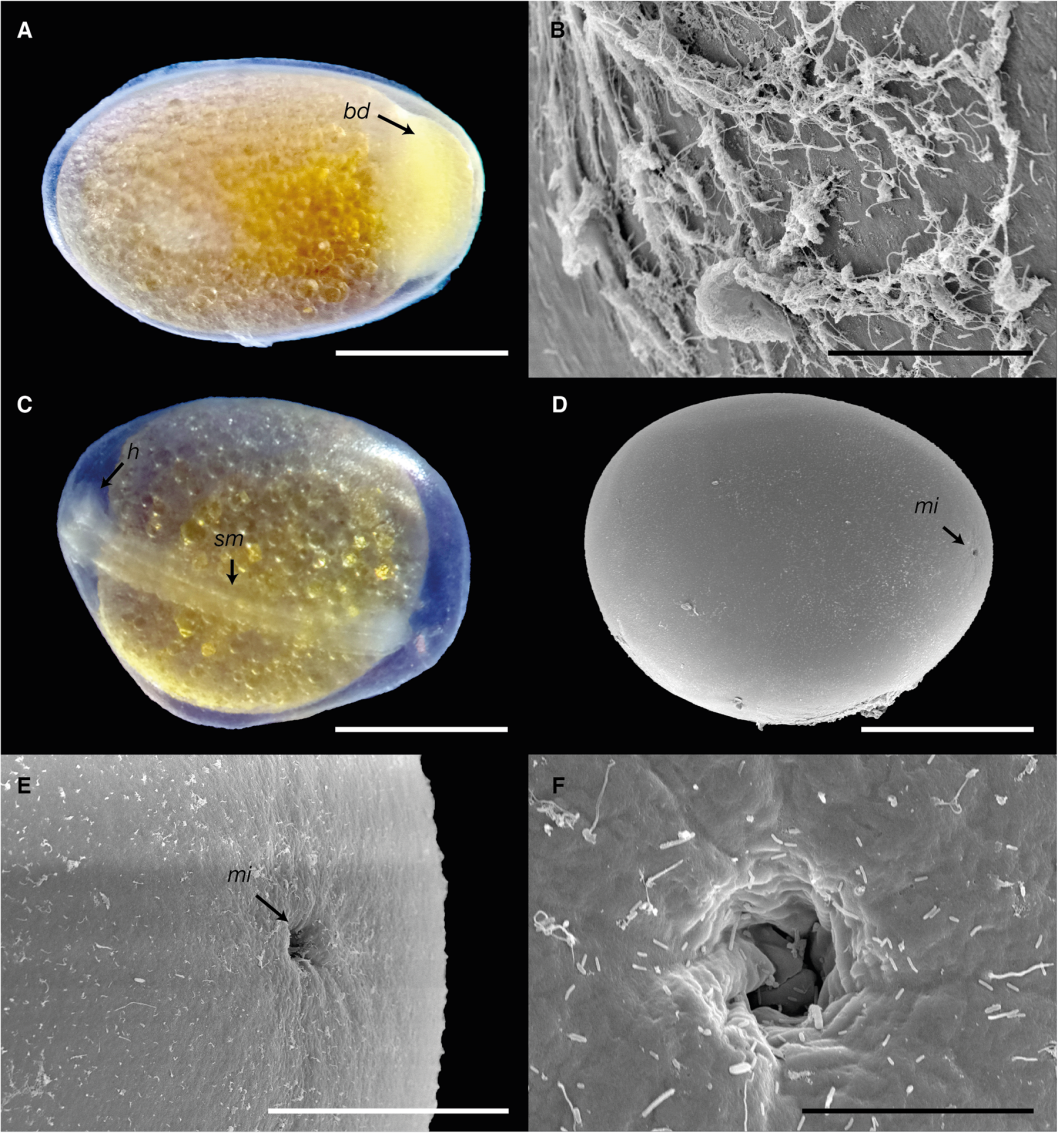
Photomicrographs and scanning electron micrographs of *Cryptoheros spilurus* eggs. (A) Zygote stage (1 hpf), scale bar = 500 μm; (B) External chorionic surface with mucous layer; scale bar = 40 μm; (C and D) 22‐somite stage (36 hpf), scale bar = 500 μm; (E) Micropylar region, scale bar = 100 μm; (F) Micropylar canal, scale bar = 20 μm. bd, blastoderm; h, head; mi, micropyle; sm, somites. [Color figure can be viewed at wileyonlinelibrary.com]

*Cryptoheros spilurus* exhibited substrate‐spawning behavior, depositing elliptical, telolecithal eggs with a *d*_*e*_ of 1.65 ± 0.05 mm (Figure 1A). The eggs, characterized by meroblastic cleavage, began hatching at 52 h post‐fertilization, with most embryos emerging by that time, although some hatched as late as 54 h. A layer of mucous secretion enveloped the eggs, facilitating their attachment to rocky substrates (Figure 1B). The micropyle, a critical structure for fertilization, displayed a distinctive funnel‐shaped morphology with a spiral ridge and measured 17.36 ± 1.03 μm in diameter (Figure 1D–F). While no specialized structures were observed in the immediate vicinity of the micropyle, the chorion in that region exhibited faint furrowed lines, suggesting subtle morphological variations in its surface texture (Figure 1E).

**Figure 2:**
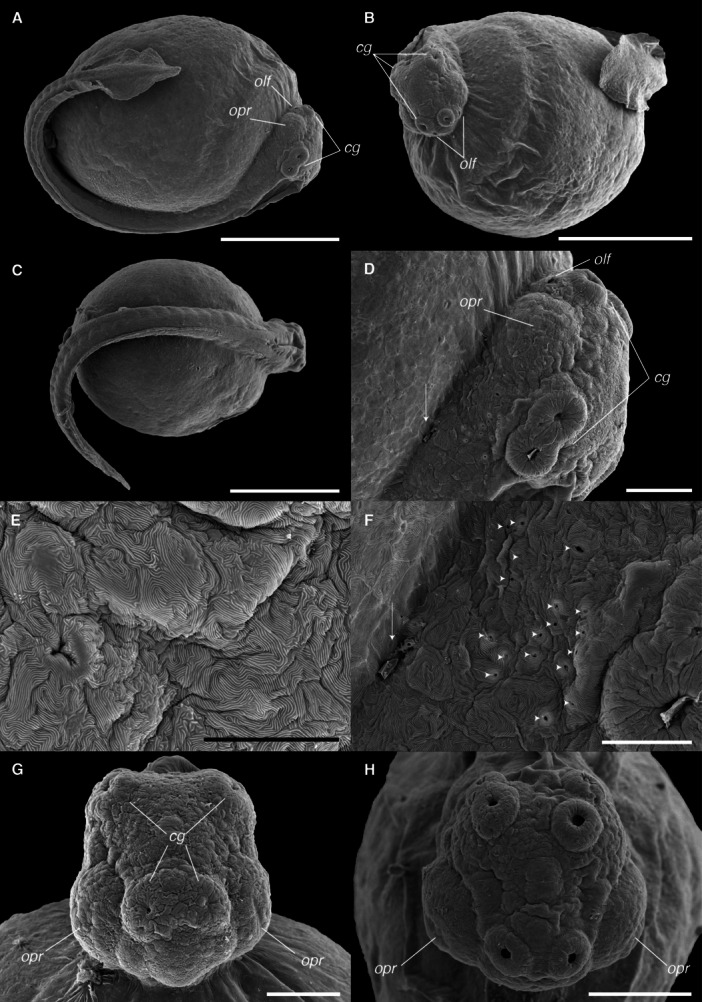
Scanning electron micrographs of *Cryptoheros spilurus* after the first hours of hatching. Overview of whole larvae at 0 hph as seen in lateral (A and B) and dorsal (C) views (scale bars = 500 μm). Lateral view of head region (0 hph) with initial gill opening (white arrow) (D; scale bar = 100 μm). Surface epithelial cells of the trunk region showing microridge patterns at 12 hph (E; scale bar = 10 μm). Surface epithelial cells of the head region showing fingerprint microridge patterns with pores of superficial goblet cells (white arrowheads) and initial gill opening (white arrow) at 0 hph (F; scale bar = 50 μm). Frontal view of the head at 12 hph (G; scale bar = 100 μm). Frontal view of the head at 24 hph (H, scale bar = 200 μm). cg, cement glands; olf, olfactory pit; opr, optic primordium.

**Figure 3:**
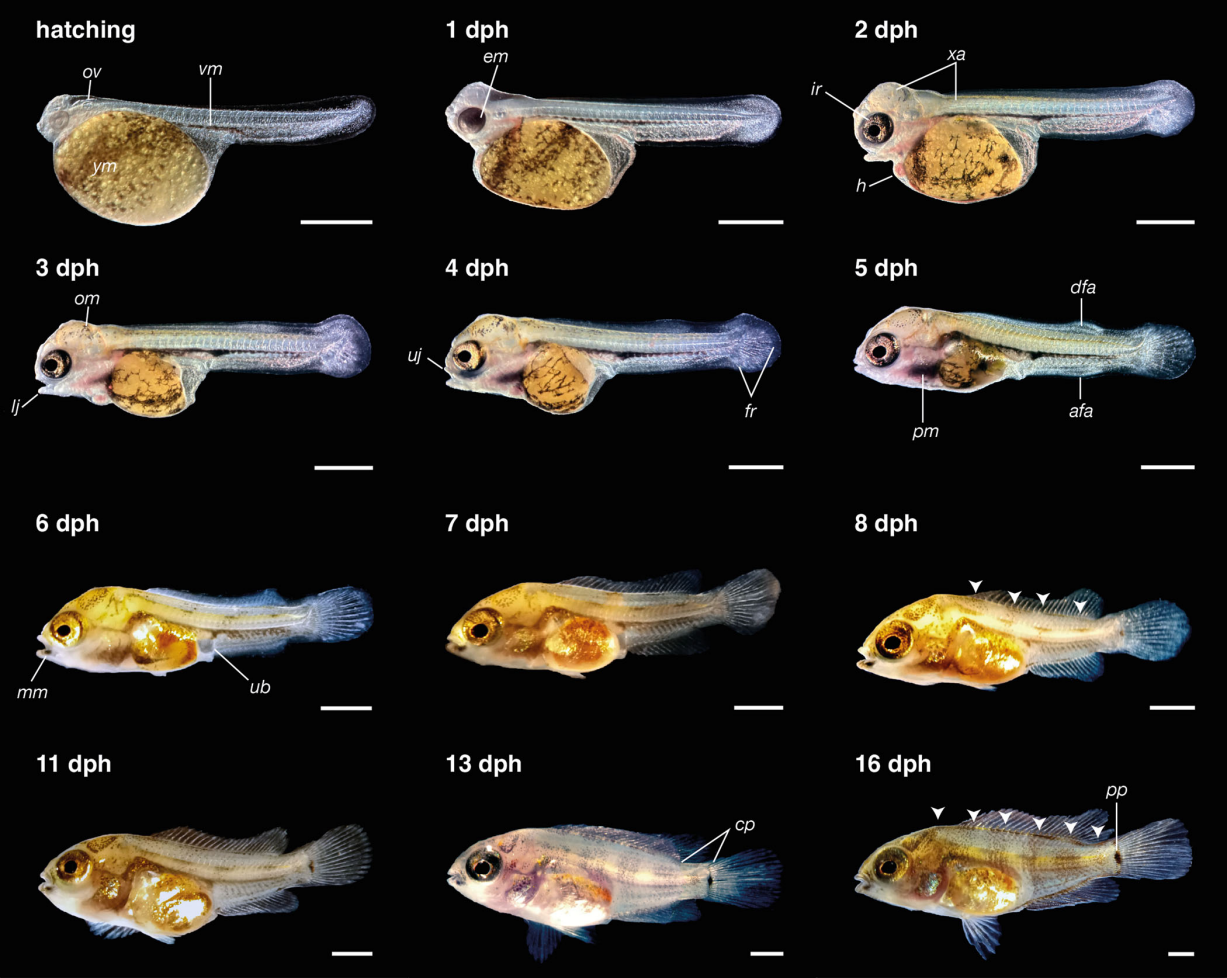
General overview of larval development in *Cryptoheros spilurus*. Representative lateral views illustrating body shape and yolk resorption at successive days post‐hatch. Dorsal blotches arranged into vertical bars (white arrowheads). afa, anal‐fin anlage; cp, caudal peduncle; dfa, dorsal‐fin anlage; em, eye melanophores; fr, fin rays; h, heart; ir, iridophores; lj, lower jaw; mm, mandibular melanophores; ov, otic vesicle; pm, pharyngeal melanophores; pp, pigment patch; ub, urinary bladder; uj, upper jaw; vm, ventral melanophores; xa, xanthophores; ym, yolk melanophores. Scale bars = 1 mm. [Color figure can be viewed at wileyonlinelibrary.com]

At hatching, *C. spilurus* larvae (TL = 4.739 ± 0.27 mm) possess a large yolk sac, a continuous finfold around the trunk and tail, a straight notochord, and poorly developed eyes (Figures 2 and 3). A ventral melanophore stripe and prominent dendritic melanophores were observed over the yolk (Figure 3). SEM images of larval external morphology immediately after hatching are presented in Figure 2, including whole‐body shots (Figure 2A–C), higher magnification shots detailing specific features such as the head region (Figure 2D), surface epithelial microridge patterns on the trunk (Figure 2E) and head (Figure 2F), and progressive changes in head morphology between 12 and 24 hph (Figure 2G–H). Key structures such as cement glands, olfactory pits, and optic primordia are already evident at these early stages (Figure 2). A general overview of larval development, including representative body shapes and yolk resorption at successive days post‐hatch, is shown in Figure 3. A detailed summary of the main developmental milestones, including total length measurements and morphological changes, is provided in Supporting Information (Table S1).

### 3.2 Head and Trunk Development

**Figure 4:**
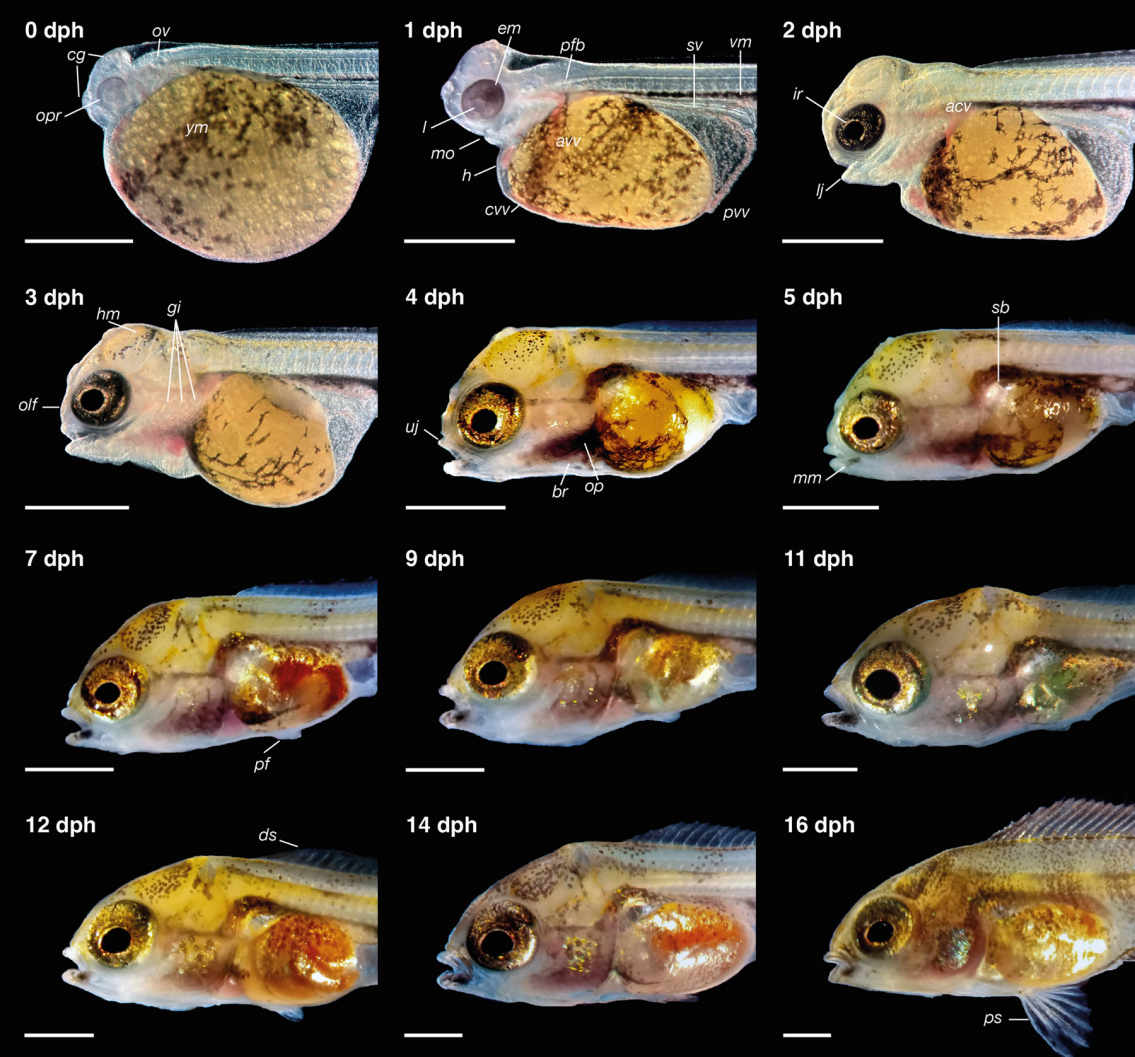
Head and trunk development in larvae of *Cryptoheros spilurus*. Lateral views of live specimens highlighting the progression of cranial structures and pigmentation patterns. acv, anterior cardinal vein; avv, anterior vitelline vein; br, branchiostegal rays; cvv, common cardinal vein; cg, cement glands; ds, dorsal spines; em, eye melanophores; g, gills; h, heart; hm, head melanophores; ir, iridophores; l, lens; lj, lower jaw; mm, mandibular melanophores; mo, mouth; olf, olfactory pit; op, operculum; opr, optic primordium; ov, otic vesicle; pf, pelvic fin; pfb, pectoral‐fin bud; ps, pelvic spines; pvv, posterior vitelline vein; sb, swim bladder; uj, upper jaw; vm, ventral melanophores; ym, yolk melanophores. Scale bars = 1 mm. [Color figure can be viewed at wileyonlinelibrary.com]

**Figure 5:**
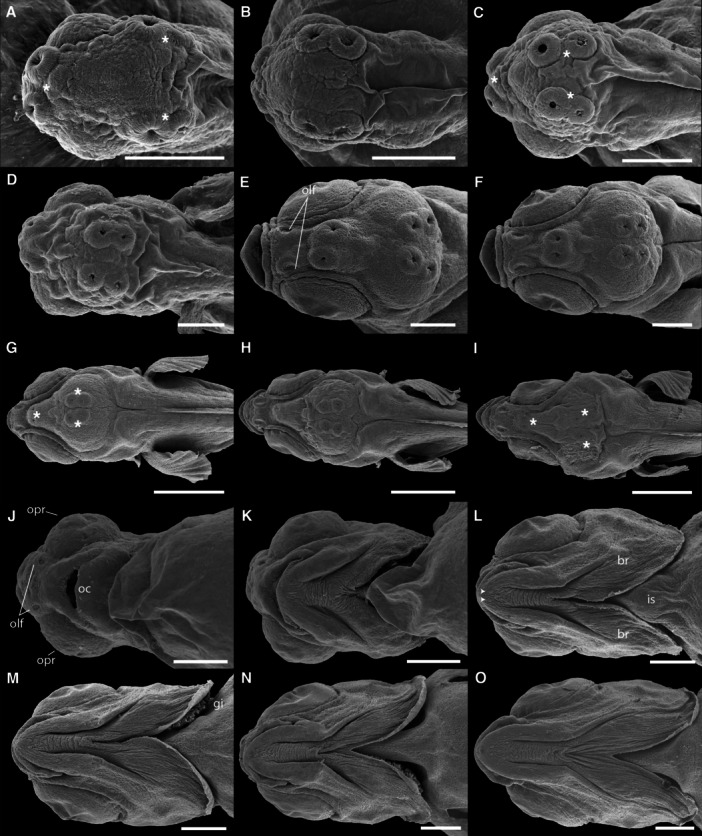
Dorsal and ventral view of head region in the early development of *Cryptoheros spilurus* larvae. SEM micrographs at 0 hph (A), 1 dph (B), 36 hph (C and J), 2 dph (D and K), 3 dph (E and L), 4 dph (F and M), 5 dph (G and N), 6 dph (H and O), and 7 dph (I). Scale bars 200 μm in A–E, J–O, and 500 μm in F–I. Cement glands indicated by white asterisks and superficial mandibular neuromasts (white arrowheads). br, branchiostegal membranes; is, isthmus; oc, oral cavity; olf, olfactory pit; opr, optic primordium.

**Figure 6:**
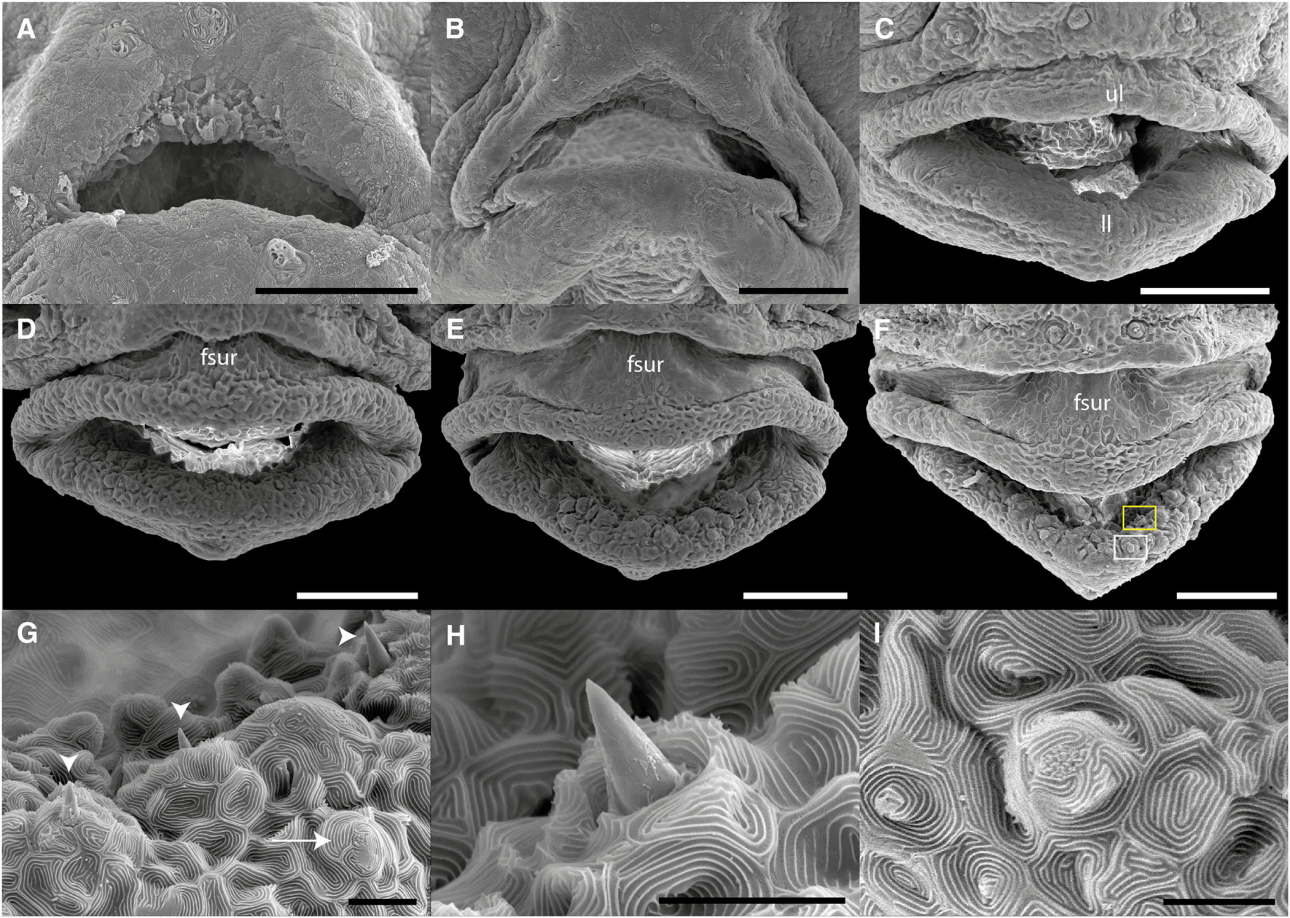
Development of the buccal cavity in *Cryptoheros spilurus* larvae. Initial mouth opening at 36 hph (A). Progressive mouth and lip development at 2, 3, 5, 6, and 7 dph, respectively (B–F). Scale bars in A–F = 100 μm. Surface architecture of the lower lip epithelium displaying teeth (white arrowheads) and taste buds (white arrow) (G). Detail of tooth with mucus secretion (yellow rectangle in F) at 7 dph (H). Higher magnification of taste bud (white rectangle in F) showing the apex with numerous packed microvilli (I). Scale bars in G–I = 10 µm. fsur, fold of skin; ll, lower lip; ul, upper lip.

The head of *C. spilurus* larvae exhibit pronounced morphological and functional differentiation during early development. As shown in Figures 4, 5, 6, notable changes in external cranial morphology occurred between hatching and 6 dph. At hatching, the head was ventrally flexed but achieved a straightened position at 1 dph. Optic primordia and lenses were visible at hatching, although unpigmented; eye pigmentation initiated by 1 dph and iridophores became evident by 2 dph (Figure 4). Otic vesicles were present in the posterior region of the head from the time of hatching. The mouth was initially closed but an open oral cavity ventrally located was observed by 36 hph (Figure 4A), with a progressive anterior migration leading to a terminal position by 3 dph (Figure 4C). Head dimensions increased steadily over time, accompanied by the differentiation of craniofacial structures. External gill openings were discernible by 12 hph; however, buccal ventilation and gill functioning was not evident until 3 dph, almost coinciding with the emergence of branchiostegal rays at 4 dph.

During the initial days post‐hatching, cranial elements such as the eyes, cement glands and olfactory pits became spatially organized, marking the early establishment of head regionalization. After 4 dph, a noticeable elongation of the head was observed, accompanied by a progressive widening that became particularly evident by 7 dph (Figure 5), reflecting both neurocranial growth and the expansion of associated sensory and skeletal structures. Branchiostegal membranes are slightly united (anteriorly) and free from the isthmus (Figure 5).

### 3.3 Buccal Cavity Development

At hatching, *C. spilurus* larvae exhibit a closed mouth. The oral cavity becomes externally visible around 36 hph, initially appearing as a simple opening without distinct jaw differentiation (Figure 6A). Over the following days, the dentary, premaxillary, and both upper (ul) and lower lips (ll) begin to form. By 3 dph, the ul and ll can be clearly distinguished, and by 6 dph, a fold of skin (fsur) connecting the dorsal head skin to the ul develops, exhibiting a visible protrusion that becomes increasingly extensible in subsequent days (Figure 6). At this stage, G‐type teeth (*sensu* Říčan et al. 2016), characteristic of detritivorous, scraping and herbivorous feeding strategies, are first observed. These teeth exhibit a pointed, conical shape with a flattened tip (Figure 6H), measuring 8.18 ± 0.61 µm in length and spaced 15–18 µm apart. Mucus secretion was visible around the teeth, and the surrounding epithelium displays a microridge pattern composed of concave depressions separated by furrows (Figure 6H). Concurrently with the appearance of teeth, multiple taste buds were observed in the buccal cavity, both externally and internally on the lips. Each taste bud forms a conical elevation from the epithelial surface, with the apex densely covered by packed microvilli (Figure 6I). GS increased from 0.396 ± 0.034 mm at 4 dph to 1.134 ± 0.057 mm at 16 dph.

### 3.4 Caudal Development

**Figure 7:**
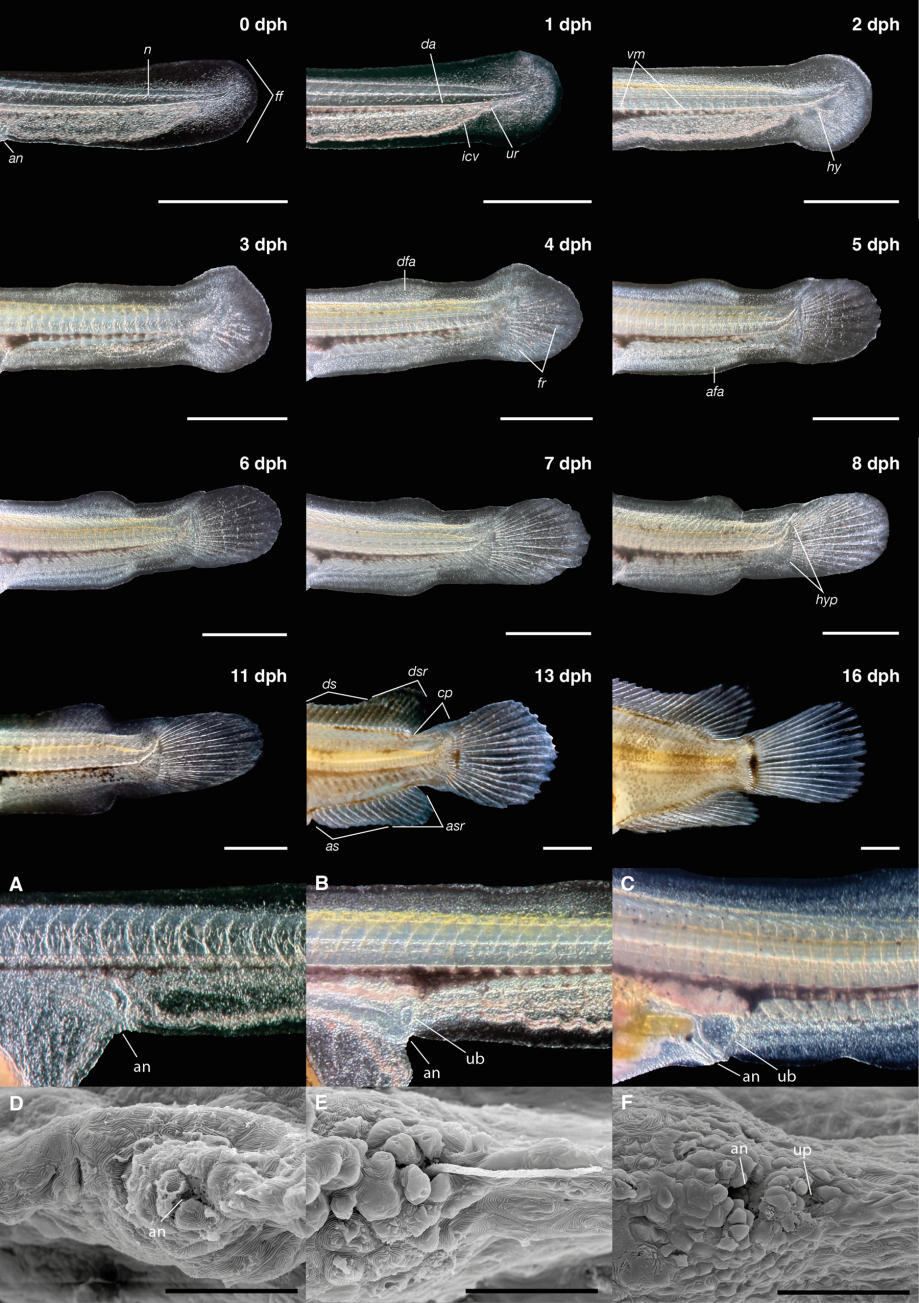
Lateral views of the caudal region in *Cryptoheros spilurus* larvae throughout development. Images show notochord flexion, caudal fin formation, and finfold regression. Scale bars = 1 mm. A–C Optical micrographs of the urogenital region (in lateral view) of a live specimen at 0 dph (A), 2 dph (B), and 7 dph (C). D–F SEM micrographs of the urogenital region (in ventral view) of larvae at 6 hph (D), 36 hph, showing excretion (E), and 3 dph (F). All specimens are oriented with the rostral end to the left. (D–F) Scale bars = 50 μm. afa, anal‐fin anlage; an, anus; as, anal spines; asr, anal soft rays; cp, caudal peduncle; da, dorsal aorta; dfa, dorsal‐fin anlage; ds, dorsal spines; dsr, dorsal soft rays; ff, finfold; fr, fin rays; hy, hypural region; hyp, hypural; icv, inferior caudal vein; n, notochord; ub, urinary bladder; up, urogenital pore; ur, urostylar artery and vein; vm, ventral melanophores. [Color figure can be viewed at wileyonlinelibrary.com]

At hatching, the notochord was straight and started to flexion by 1 dph with the end of the flexion at 5 dph (Figure 7). Since hatching, a strong vascularization was visible in the ventral finfold, decreasing in subsequent days (Figure 7). The terminal portion of the digestive tract was visible from the time of hatching, and the anal opening was already formed at this stage (Figure 7A,D). The urinary bladder became distinguishable by 2 dph (Figure 7B) and continued to increase size during subsequent days of development (Figure 7C). Evidence of excretory activity was observed as early as 36 hph (Figure 7E), and by 3 dph, the urogenital pore could be clearly differentiated from the anal opening (Figure 7F).

### 3.5 Fin Development

At hatching (TL 4.74 ± 0.27 mm), larvae possessed a continuous median finfold, a membranous structure extending dorsally from the head–trunk junction along the dorsal trunk and ventrally from the anus, encircling the posterior tip of the tail (Figures 2, 3, 7). The first evidence of fin differentiation was observed in the caudal fin region, coinciding with the initial flexion of the notochord at 2 dph (TL 5.76 ± 0.21 mm), when the posterior finfold expanded and began to form a rounded lobe. By 3 dph (TL 5.86 ± 0.26 mm), the dorsal portion of this structure protruded conspicuously, aligning with the axis of notochord flexion. At this stage, the first lepidotrichia were visible, and the fin appeared slightly longer ventrally than dorsally. However, by 5 dph (TL 6.37 ± 0.13 mm), symmetry was achieved between the dorsal and ventral caudal lobes, coinciding with free‐swimming. Ray segmentation in the caudal region was evident by 4 dph (TL 6.19 ± 0.23 mm), and fin development proceeded steadily thereafter. By 14 dph (TL 10.97 ± 0.46 mm), all median fins had developed their definitive complement of skeletal elements.

Development of the dorsal and anal fins followed in a sequential pattern. In the dorsal region, a localized bulging of the finfold, indicative of the dorsal‐fin anlage, was first noted at 3 dph (Figure 7). The first visible dorsal fin rays appeared at 6 dph (TL 6.62 ± 0.25 mm), and segmentation progressed gradually until complete formation of the dorsal fin, including both segmented and unsegmented rays, was achieved at 14 dph, and caudal peduncle was evident. Similarly, the anal‐fin primordium was distinguished by a prominent ventral bulge posterior to the anus around 5 dph (TL 6.37 ± 0.13 mm). Lepidotrichia became discernible at 9 dph (TL 7.89 ± 0.26 mm), and full development of the anal fin occurred by 14 dph, paralleling the completion of the dorsal fin.

**Figure 8:**
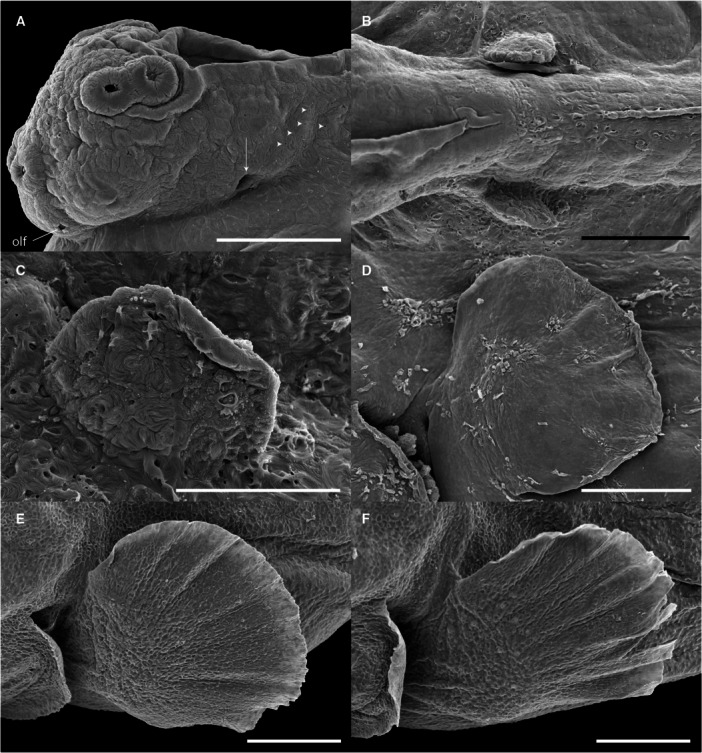
Scanning electron micrographs of pectoral fin development of *Cryptoheros spilurus*. Lateral view of head region at 12 hph without the presence of pectoral fin bud and showing external gill opening (white arrow) and pores (white arrowheads) (A; scale bar = 200 μm). Dorsal view of trunk region at 1 dph where pectoral fin buds are observed (B; scale bar = 200 μm). Lateral view of pectoral fin bud at 1 dph (C; scale bar = 100 μm). Lateral view of pectoral fin at 3 dph (D), 5 dph (E), and 6 dph (F). Scale bars in D–F = 200 μm. olf, olfactory pit.

The paired pelvic fins developed later. Small‐paired buds became visible ventrolaterally at 6 dph (TL 6.62 ± 0.25 mm), located posterior to the pectoral region and near the developing posterior swim bladder lobe. The first fin rays emerged at 12 dph (TL 9.06 ± 0.39 mm), with full development, including segmented and unsegmented rays, attained by 16 dph (TL 13.17 ± 0.55 mm). In contrast, the pectoral fins exhibited precocious development. Fin buds were already evident shortly after hatching at 1 dph (TL 5.21 ± 0.20 mm) and continued to grow steadily (Figure 8). Fully formed pectoral fins, with segmented rays and well‐delineated margins, were observed at 7 dph (TL 6.95 ± 0.23 mm).

By the end of metamorphosis, all fins were fully formed and their elements clearly distinguishable under light microscopy and after clearing and staining. The anal fin presented 9 spines and 7 soft rays, totaling 16 elements, while the dorsal fin exhibited 18 spines and 9 soft rays, for a total of 27 elements. These counts were consistent across all specimens examined. The caudal fin displayed a rounded shape supported by 18 principal rays, along with 6 peripheral procurrent rays (3 dorsal and 3 ventral; Figure 7). Additionally, the pectoral fins contained 14 rays, and the pelvic fins were supported by 5 rays.

### 3.6 Cement Glands

**Figure 9:**
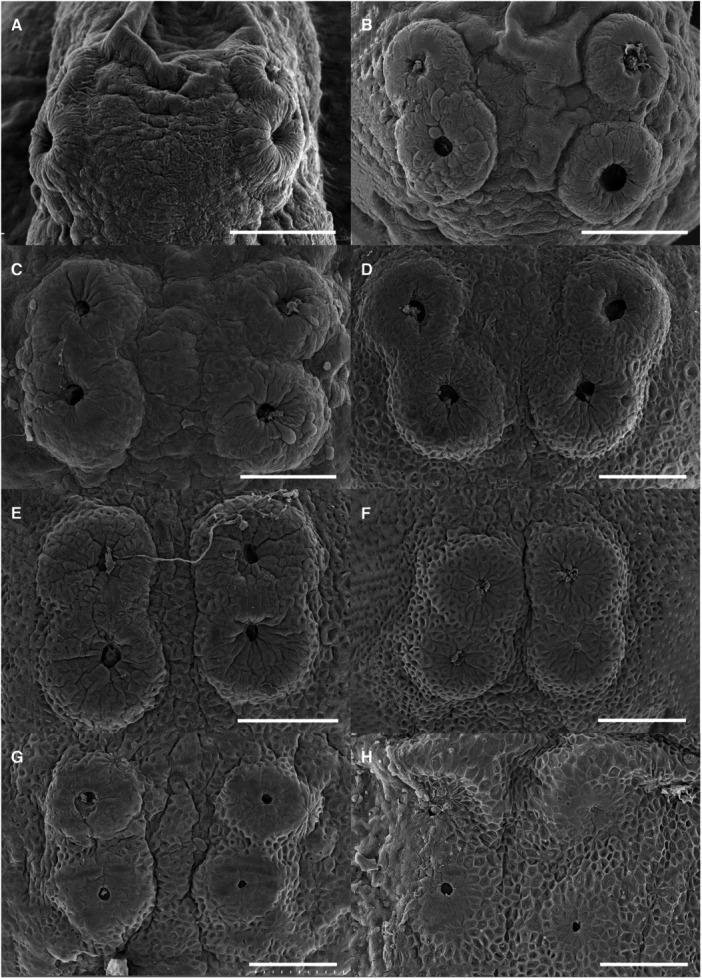
Scanning electron micrographs of dorsal cement glands in the parietal area of *Cryptoheros spilurus* during larval development. At 0 dph (A), 1 dph (B), 2 dph (C), 3 dph (D), 4 dph (E), 5 dph (F), 6 dph (G), and 7 dph (H). Scale bars = 100 μm.

**Figure 10:**
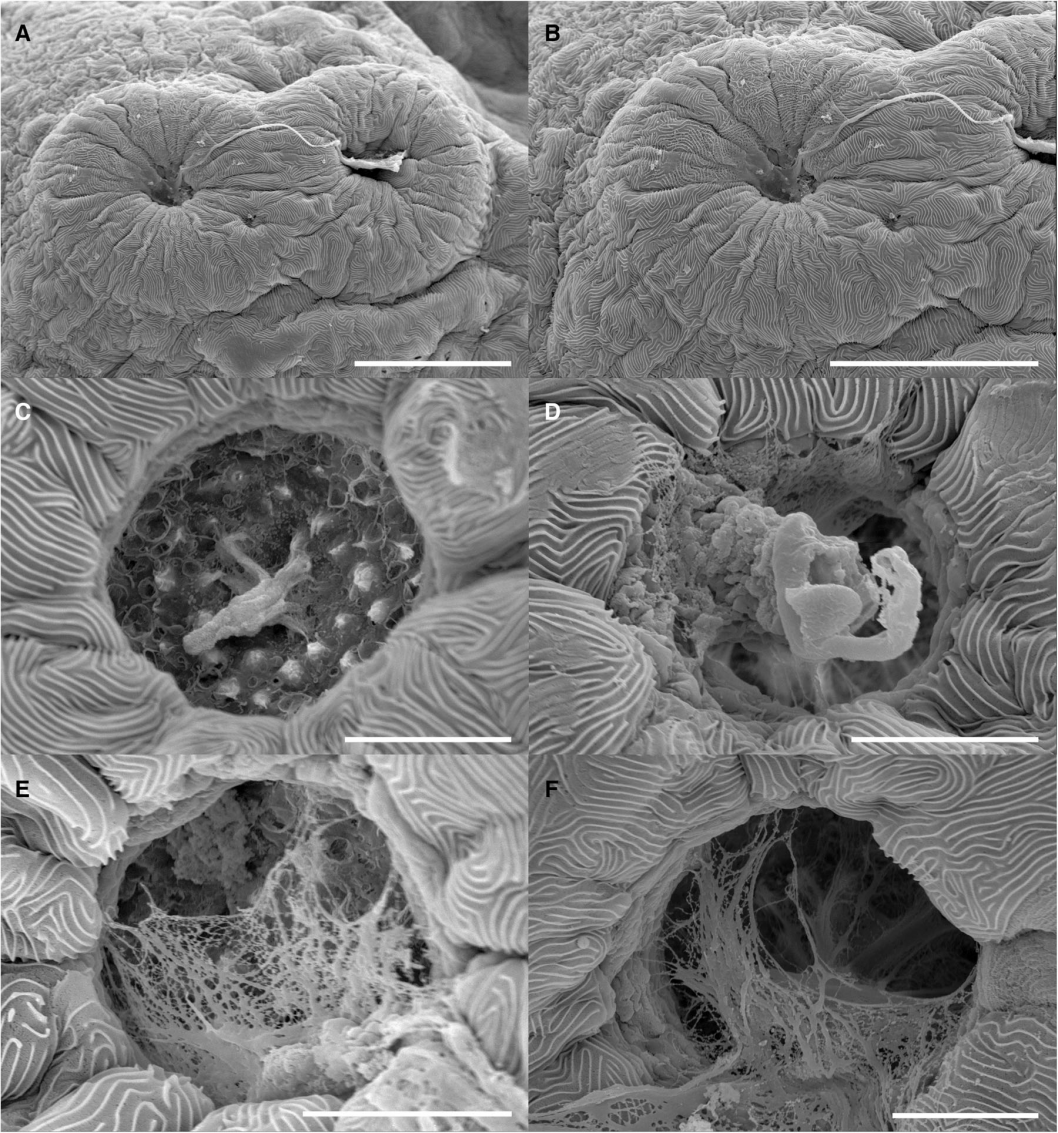
Scanning electron micrographs of cement glands of *Cryptoheros spilurus*. Pair cement glands at 0 hph (A–B; scale bars = 50 μm). Closeup of cement glands showing numerous microvilli at 1 dph (C–D) and mucous secretion at 2 dph (E–F); scale bars = 10 μm.

Cement glands are adhesive mucus‐secreting organs observed in early larval stages of substrate‐brooding fishes. At hatching, the cement gland apparatus consisted of three pairs of prominent glandular elevations on the larval head. Two pairs were located dorsally in the parietal region, just above the eyes, and the third was situated frontally, near the olfactory structures (Figures 2, 5, and 9). The positional dynamics and regression of the parietal pairs are illustrated in Figure 9. Ultrastructural details and evidence of mucus secretion are presented in Figure 10. The cement glands appeared as crateriform glandular domes (Figure 10A–B), approximately 100 µm in diameter, characterized by a central pore with numerous microvilli (Figure 10C) and conspicuous radial surface ridges. These dome‐like structures secreted adhesive mucus through their apical pores (Figure 10C–F), facilitating transitory larval attachment. Although the size of the glands remained relatively stable throughout the first post‐hatching days, their spatial arrangement changed progressively. A medial migration of the parietal pairs was observed, bringing them in close proximity, though not complete fusion, by 5 dph (Figures 5G and 9F) coinciding with emergence of free swimming. Subsequently, the glands began to regress, becoming flattened and barely elevated by 7 dph (Figures 5I and 9H).

### 3.7 Sensory Organs

**Figure 11:**
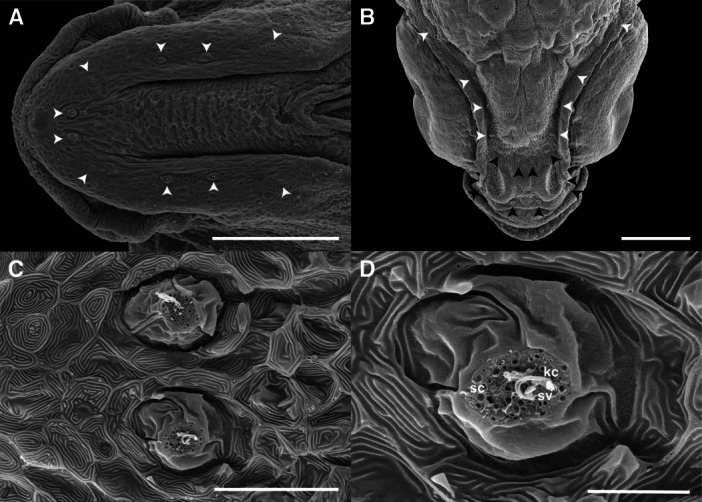
Distribution and ultrastructure of superficial neuromast in *Cryptoheros spilurus* larvae. Ventral view showing the superficial mandibular neuromasts (white arrowheads) at 6 dph (A). Dorsal view showing the distribution of supraorbital neuromast (white arrowheads) and supranasal neuromast (black arrowheads) (B). Morphology of a round‐shaped superficial mandibular neuromast at 4 dph (C and D). Scale bars = 200 μm in A and B, 30 μm in C, and 10 μm in D. kc, kinocilium; sc, non‐sensory support cells, sv, stereovilli.

The lateral line system in cichlids is a crucial sensory system for detecting movements and vibrations in the water. Its functional units are neuromasts, mechanoreceptors that allow these fish to perceive changes in their aquatic environment. In the cranial region of *C. spilurus* larvae, superficial neuromasts (SNs) were observed embedded in the epidermis. Dorsal and ventral views of the head are presented in Figure 11, illustrating the spatial distribution of these neuromasts. The first SNs appeared by 3 dph, with an approximate diameter of 20 µm. Dorsally, the neuromast were positioned in the supraorbital (SO) and supranasal (SUN) regions, whereas ventral neuromasts were localized in the superficial mandibular (SM) area (Figure 11A–B). These early‐stage neuromasts consisted of a round macula composed of centrally located hair cells, each bearing a kinocilium and multiple stereocilia (sterovilli), surrounded by non‐sensory support cells (Figure 11C–D). The sensory hair bundles were exposed at the epithelial surface, indicating their classification as SNs.

**Figure 12:**
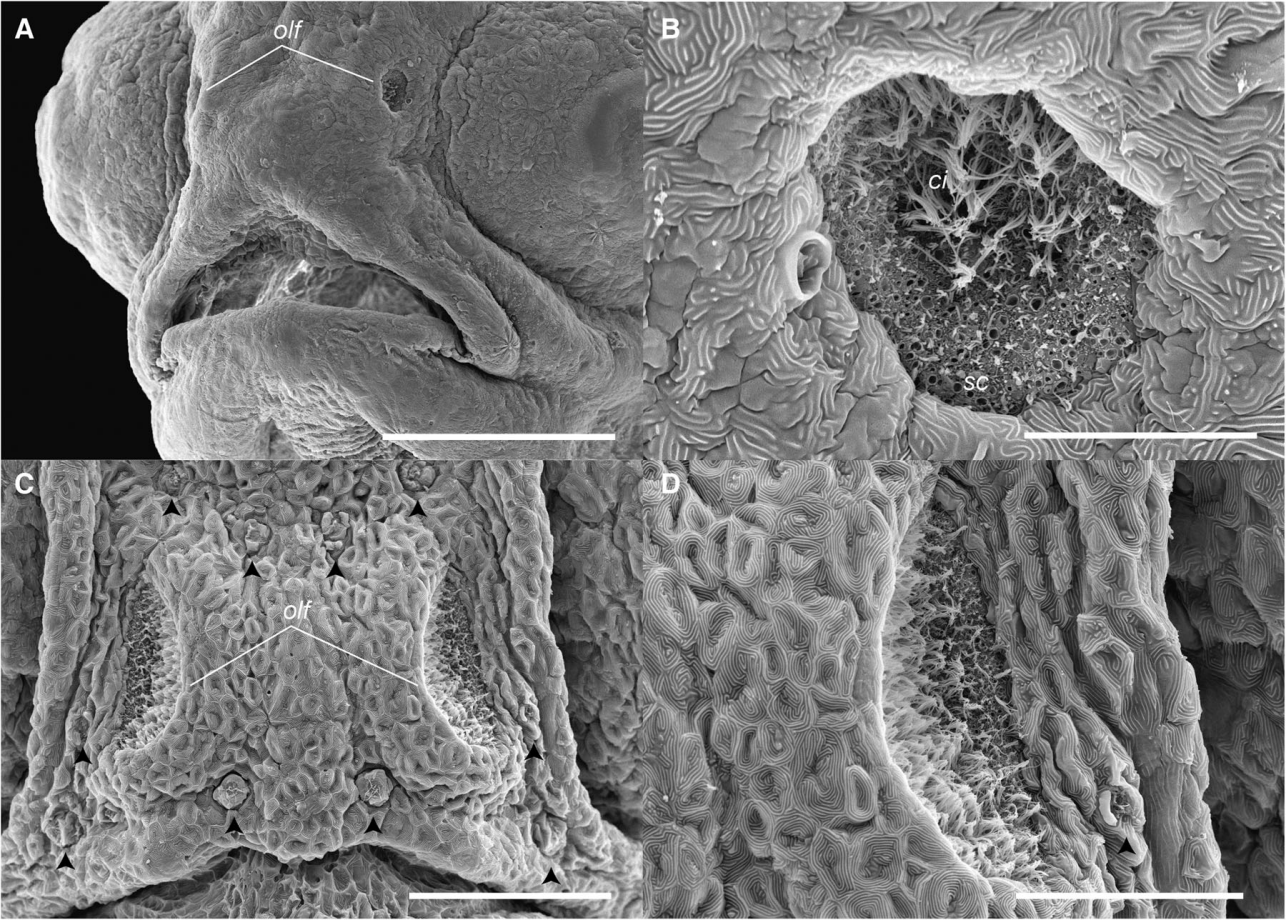
Development of olfactory pit in *Cryptoheros spilurus*. Head region at 2 dph (A; scale bar = 200 μm). Olfactory pit at 2 dph (B; scale bar = 20 µm). Frontal view of olfactory pits and supranasal neuromast (black arrowheads) at 5 dph (C and D); scale bar = 100 µm in C and 50 µm in D. ci, cilia; sc, non‐sensory supporting cells; olf, olfactory pit.

The olfactory pit was visible immediately after hatching (Figure 2A–B,D) as an ellipsoidal invagination characterized by a shallow depression densely packed with cilia. Over time, the pit progressively deepened, ranging from approximately 10 µm at 0 dph to 92 ± 3.11 µm at 7 dph (Figures 5E–I, 12).

### 3.8 Pigmentation

Pigmentation in *C. spilurus* larvae exhibited progressive regionalization and diversification throughout early development. At hatching, larvae displayed a ventral melanophore stripe (vm) and large dendritic melanophores over the yolk sac (ym; Figure 3). By 2 dph, xanthophores emerged in the cranial region and along the dorsal midline, while iridophores (ir) became visible within the iris (Figure 4). Occipital melanophores (om) appeared spreading (Figure 3), and the ventral stripe became more condensed, especially above the terminal portion of the gut (Figure 7). At 3 dph, the eyes exhibited complete pigmentation, and both occipital melanophores and cranial xanthophores increased in number and intensity. Melanophores also developed beneath the pharyngeal region (pm = pharyngeal melanophores; Figure 3), and scattered melanophores began to appear laterally along the trunk and tail (Figure 7). By 5 dph, the ventral stripe extended from the posterior edge of the swim bladder to the tail, accompanied by dispersed melanophores in the peritoneal and ventrolateral areas (Figure 3). The pharyngeal melanophore patch became more prominent, and mandibular melanophores emerged (mm). At 6 dph, both the lateral and ventral stripes exhibited a dashed appearance and began transitioning into a vertical arrangement by the completion of metamorphosis (16 dph), along with the emergence of dense pigment patch (pp) in the basicaudal region (Figure 3).

### 3.9 Ontogenetic Allometry in *Cryptoheros spilurus*

**Figure 13:**
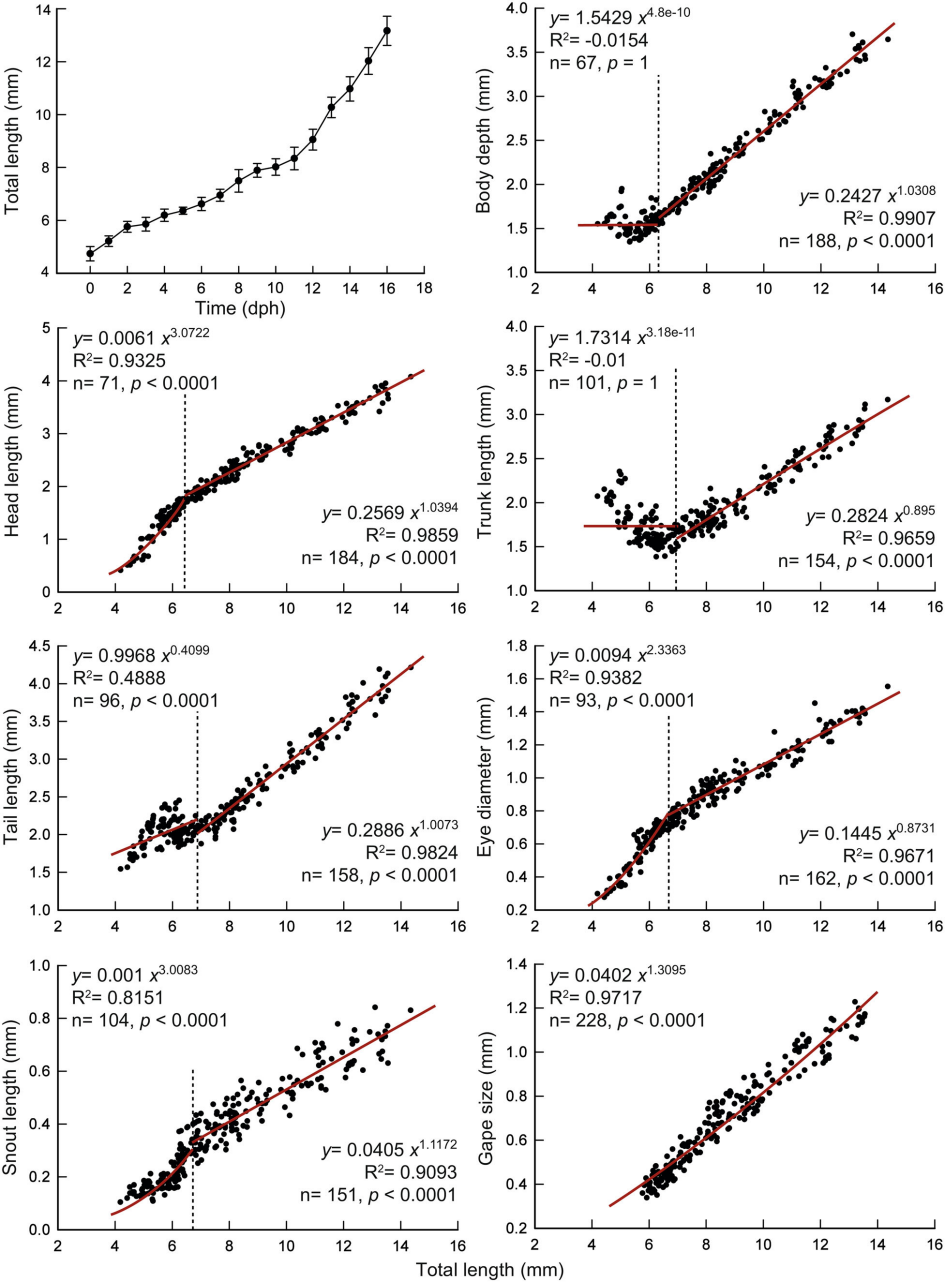
Growth curve and allometric growth patterns of *Cryptoheros spilurus* during larval development. Growth curve is based on total length (TL, mm) measurements of live specimens, each data point represents the mean TL of 15 larvae, with vertical bars indicating standard deviation. Scatter plots showing the relationship between total length (TL) and each measured body proportion: tail length (TAL), trunk length (TRL), head length (HL), eye diameter (ED), snout length (SNL), body depth (BD), and mouth gape size (GS). Observations are shown as dark points. Red lines represent the fitted nonlinear models (power functions), and dashed vertical lines indicate the inflection points where changes in allometric growth patterns occur. GS did not exhibit an inflection point and is represented with a single model. All models were statistically significant (*p* < 0.05), except for post‐inflection TAL (*p* = 0.6294), which showed isometric growth. [Color figure can be viewed at wileyonlinelibrary.com]

The growth trajectory is shown in Figure 13, with TL mean values and associated variability represented by standard deviation. A steady increase in TL was observed throughout the period, with a marked acceleration by 11 dph. The morphological proportions of *C. spilurus* exhibited distinct allometric growth patterns during larval development (Figure 13). Most traits presented a growth inflection point, except for GS, which showed a consistent positive allometric pattern throughout the analyzed period (*b* = 1.3095; *p* < 0.0001). Before the inflection point, negative allometric growth was observed for TAL, TRL, and BD (*b* = 0.4099, 3.18 × 10^−11^, and 4.81 × 10^−10^, respectively; *p* < 0.0001), while HL, ED, and SNL exhibited positive allometry (*b* = 3.0722, 2.3363, and 3.0083, respectively; *p* < 0.0001). After the inflection point, HL and SNL remained positively allometric (*b* = 1.0394 and 1.1172; *p* = 0.00279 and 0.00572, respectively) and BD transitioned to positive allometry (*b* = 1.0308; *p* = 0.00316). TRL maintained negative allometric growth (*b* = 0.895; *p* < 0.0001) and ED shifted to a negatively allometric pattern (*b* = 0.8731; *p* < 0.0001). TAL displayed isometric growth following the inflection point (*b* = 1.0073; *p* = 0.62945). Reductions in growth coefficients were evident for HL, ED, and SNL after the inflection point (*b* = 3.0722 vs. 1.0394; 2.3363 vs. 0.8731; 3.0083 vs. 1.1172, respectively).

## 4 Discussion

The early development of *Cryptoheros spilurus* illustrates a larval phase characterized by transient morphological and physiological features, that reflects both phylogenetic constraints and ecological adaptations, core themes in evolutionary developmental biology. As in other substrate‐brooding Neotropical cichlids, development is characterized by rapid post‐hatching differentiation and early exogenous feeding, but *C. spilurus* displays some derived features that underscore lineage‐specific modifications of conserved developmental programs. Cement glands, known from other substrate‐spawning cichlids, are present as three pairs of adhesive structures involved in larval aggregation and parental care; however, our study provides a detailed account of their chronological development and regression, documenting their transient deployment within a narrow temporal window. This developmentally transient feature exemplifies modular morphological innovation and loss within early ontogeny. Additional transient traits, such as the respiratory plexuses and continuous median finfold, support physiological function during early stages and are gradually replaced by definitive juvenile structures through coordinated morphogenesis. A pronounced inflection in allometric growth and pigmentation trajectories further indicates a modular developmental shift marking the larvae‐to‐juvenile transition. These findings position *C. spilurus* as a useful system for examining the evolution of developmental timing (heterochrony), structural modularity, and life‐history transitions across closely related cichlid species. While some observed traits may reflect adaptation to specific environmental conditions, others likely represent conserved developmental patterns among teleosts, emphasizing the need for comparative data to disentangle constraint from adaptation.

Given its use of complex habitats and variable substrates, early fin development in *C. spilurus* is expected to be crucial for survival. Fin development in fishes is a complex and highly regulated process essential for locomotion, stability and other vital functions (Meijide and Guerrero 2000; Osse and Van den Boogaart 2004; Kupren et al. 2014). It begins with the formation of a median finfold, a temporary larval structure that extends dorsally, caudally, and ventrally along the body (Figures 2 and 3). Individual fins develop sequentially, beginning with mesenchymal condensation, followed by cartilage formation and subsequent ossification (Parichy et al. 2009). The timing and pattern of fin development vary among species, reflecting adaptations to different ecological and evolutionary pressures. For instance, in *Cichlasoma dimerus*, the pectoral fin is the first to show cartilage structures (Meijide and Guerrero 2000; Beriotto et al. 2023), while in zebrafish, the pectoral fins develop in embryogenesis, and the caudal fin develops after hatching before the anal and dorsal fins (Parichy et al. 2009). The pectoral fins in *C. spilurus* exhibited precocious development, while pelvic fins appeared later in the development. The first evidence of fin differentiation in the finfold appeared in the caudal region at 2 dph, coinciding with the initial notochord flexion and the expansion of the posterior finfold into a rounded lobe. Ray formation became evident by 3 dph, and symmetry between dorsal and ventral lobes was achieved by 5 dph. The rapid development of the caudal fin suggests an early functional need for efficient propulsion, consistent with survival in dynamic aquatic environments. Dorsal and anal fins followed a coordinated developmental pattern. Starting with a bulging of the dorsal finfold, signaling the dorsal fin anlage, appeared at 3 dph, with segmented dorsal rays visible by 6 dph and full formation by 14 dph. Similarly, the anal fin primordium developed by 5 dph, with lepidotrichia appearing at 9 dph and completion at 14 dph. Ecologically, fins are essential innovations enabling fishes to diversify into various niches (Karachle and Stergiou 2012; Schneider et al. 2023). Given the variability of substrates, the presence of early fin development and cement glands may significantly increase the probability of successful attachment and locomotion, which is essential for survival in fluctuating habitats.

Cement glands are transient, mucus‐secreting structures found in the early larval stages of several teleosts and amphibians, facilitating substrate attachment (Pottin et al. 2010; Maurakis and Maurakis 2017). In *C. spilurus*, six dome‐shaped glands (two frontal and four parietals) are located on the anterodrosal region of the head (Figures 2, 5, and 9). These glands, which secrete adhesive mucus, aid larvae in anchoring to substrates during the first few days post‐hatching. The parietal glands move medially to form a central group by 5 dph, but do not fuse completely (Figure 9). They regress and are nearly undetectable by 7 dph (Figure 9H), consistent with other neotropical cichlids (Molina‐Arias 2011; Kratochwil et al. 2015; Contreras‐Tapia et al. 2024b). This regression aligns with the transition from substrate attachment to independent swimming. Cement glands contribute to energy conservation and predator avoidance by anchoring larvae to stable substrates (Groppelli et al. 2003), with trigeminal innervation enabling a rapid response to stable surfaces (Mabee et al. 1998; Pottin et al. 2010). In *C. spilurus*, cement glands are adaptative for substrate‐breeding species in moderate‐flow environments, reducing displacement and facilitating post‐hatching parental care (Buege et al. 2021). From a comparative perspective, cement glands exhibit significant interspecific variability in number, morphology, and persistence, reflecting ecological and evolutionary divergence. In fast‐flowing environments, species like *Devario malabaricus* retain adhesive glands for up to 2 weeks to secure both eggs and larvae (Britz et al. 2000; Nelson et al. 2019). Conversely, mouth‐brooding cichlids, which provide internal protection, lack cement glands entirely (Fujimura and Okada 2007; Pottin et al. 2010; Saemi‐Komsari et al. 2018). This loss underscores the influence of reproductive strategy on the presence or absence of these structures (Pottin et al. 2010; Hall 2012; Nelson et al. 2019). Although cement glands have been reported in other substrate‐spawning cichlids, our study provides, for the first time, a detailed developmental chronology of their regression, reinforcing their role as transient larval features consistent with indirect development *sensu* Balon (1999).

While cement glands enable attachment to stable substrates during the early life stages, neuromasts play an equally important role in helping larvae detect hydrodynamic cues from their environment, further enhancing their ability to survive and navigate. Neuromasts in *C. spilurus* increase in number during early larval development. These mechanosensory organs, part of the lateral line system, detect water flow, pressure gradients, and hydrodynamic disturbances (Becker et al. 2016; Otsuka et al. 2023; Webb 2023). Neuromasts contain hair cells with stereocilia and a kinocilium, and non‐sensory supporting cells (Schmitz et al. 2008; Becker et al. 2016). SNs positioned directly on the skin surface are primarily responsive to water velocity, whereas canal neuromasts, located within subdermal canals, are more sensitive to pressure gradients and water acceleration (Becker et al. 2016; Majoris et al. 2021; Webb 2023). The orientation of hair cells within a neuromast determines its axis of sensitivity. Some neuromasts exhibiting bidirectional polarity and others radial polarity, the latter potentially allowing larval fish to detect multiaxial stimuli. This capacity is essential for ecologically relevant behaviors such as prey detection, prey capture, predator avoidance, schooling, navigation, and rheotaxis (Bird and Webb 2014; Becker et al. 2016; Webb 2023). Due to their exposed location and ease of access, SNs serve as valuable model for studying phenotypic variation in peripheral sensory systems, as well as the genetic and molecular mechanisms underlying hair cell differentiation, polarity, and regeneration (Schmitz et al. 2008; Bird and Webb 2014; Becker et al. 2016; Webb 2023). Nonetheless, despite their ecological and evolutionary significance, the development, genetic regulation, and adaptative significance of neuromasts in Neotropical cichlids remain largely unexplored. Future research on the early development of neuromasts in *C. spilurus* could therefore expand our knowledge of the functional and evolutionary diversity of lateral line systems and, in turn, contribute to bridge the gap between our understanding of sensory biology and ecology in fishes.

Ontogenetic allometry in *C. spilurus* shows that key inflection points in the allometric trajectories occur around 5–6 dph, coinciding with the onset of the free‐swimming stage and exogenous feeding. This shift suggests a coordinated change in growth priorities driven by increased locomotor activity and the functional demands of independent foraging. Notably, HL remains positively allometric throughout early development, in contrast to typical ontogenetic trajectories observed in many teleost fishes, where cranial growth commonly transitions from initial positive allometry to isometric or even negative allometry during later development (Osse et al. 1997; Kupren et al. 2014). The continued enlargement of the head reflects ongoing functional demands shaped by ecological and evolutionary pressures. This positive allometric growth of the head aligns with widely recognized developmental priorities in teleosts: the rapid formation of the brain, eyes, olfactory organs, branchial arches, and oral jaws (Van Snik et al. 1997; Gisbert 1999; Osse and Van den Boogaart 2004). These structures are essential for feeding, respiration, and environmental sensing, crucial functions during the transition from endogenous yolk feeding to active, exogenous foraging. Ultrastructural observations further support this developmental emphasis, revealing the maturation of neurosensory and muscular systems involved in orientation and feeding. From a structural perspective, extended cranial growth may also be attributed to delayed ossification patterns or the late development of muscle attachment sites, as observed in other cichlids (Beriotto et al. 2023). This ontogenetic strategy suggests that *C. spilurus* is adapted to a niche requiring early and persistent cranial specialization (Hulsey et al. 2019), with potential benefits for growth, survival, and competitive success.

This positive allometry may also reflect adaptations associated with the development for specialized jaw musculature in species for which plants and algae constitute the primary food source. In contrast to piscivorous or zooplanktivorous cichlids where cranial growth supports the capture of mobile prey (Karachle and Stergiou 2012; Becker et al. 2016), the continued expansion in this predominantly herbivorous species may facilitate the development of robust jaw musculature and specialized oral and pharyngeal structures for processing plant material (Sibbing and Witte 2005; Cochran‐Biederman and Winemiller 2010; Conith et al. 2019; Hulsey et al. 2019). The positive allometric growth of mouth GS during early development further supports this interpretation. An enlarged gape likely enhances the ability to access to structurally complex food sources, such as benthic algae or macrophyte fragments (Cochran‐Biederman and Winemiller 2010), increasing the bite area and improving contact with epilithic algae or macrophyte fragments (resources previously described for *C. spilurus;* Cochran‐Biederman and Winemiller 2010; Buege et al. 2021). Although *C. spilurus* is generally considered omnivorous during juvenile and adult stages, exhibiting a predominantly plant‐based diet with a secondary tendency toward invertebrate consumption (Valtierra‐Vega and Schmitter‐Soto 2000; Conith et al. 2019; Hulsey et al. 2019), it is likely that larvae initially feed on zooplankton during the early days of exogenous feeding. This pattern is widely observed among teleosts, as newly hatched larvae possess an undeveloped gastrointestinal tract composed of a simple tube with a single loop and limited digestive functionality (Day et al. 2011). Consequently, fish larvae are functionally carnivorous at early stages and rely on zooplankton in the wild until their gastrointestinal tract becomes fully differentiated (Krogdahl et al. 2011), in agreement with the long gut morphology observed in adult *C. spilurus* (Cochran‐Biederman and Winemiller 2010).

A distinct biphasic growth pattern was observed in ED marked by an initial phase of positive allometry followed by negative allometry after the inflection point. This shift reflects a common developmental trend in teleosts, where visual structures are prioritized early for efficient foraging and predator detection (Van Snik et al. 1997; Gisbert 1999; Osse and Van den Boogaart 2004; Yang et al. 2020). Enhanced visual acuity is particularly advantageous in complex or high‐flow environments, such as those inhabited by *C. spilurus* (Buege et al. 2021). Similar patterns have been documented in *Vieja fenestrata* (Middel American/Neotropical), *Labidochromis caeruleus* (Lake Malawi radiation), and *Nannacara anomala* (South American/Neotropical), where eye grows during early development to improve environmental awareness (Kupren et al. 2014; Saemi‐Komsari et al. 2018; Contreras‐Tapia et al. 2024b). After the inflection point, the reduced relative growth of ED likely reflects a shift in energy allocation toward other structures, such as the trunk and fins, as larvae improve their locomotor competence (Osse and Van den Boogaart 2004).

The negative allometric growth of TRL, TAL, and BD before the inflection point indicates that these body regions were not prioritized during early development, in favor of structures supporting immediate survival (Osse et al. 1997; 2004). This pattern is consistent with other teleosts, where trunk region growth accelerates only after initial investment in the head and sensory organs (Osse & Van den Boogaart 2004). In particular, TRL displayed persistent negative allometry throughout the larval period, suggesting a swimming mode that does not rely on trunk elongation (Osse & Van den Boogaart 2004; Huang et al. 2021). Furthermore, the presence of a yolk sac may limit trunk expansion before its resorption (Contreras‐Tapia et al. 2024b). In contrast, BD exhibited a shift to positive allometry after the inflection point, possibly corresponding to the internal organ development and improved arrangement (Peña and Dumas 2009; Saemi‐Komsari et al. 2018). As in other species, this shift may be linked to the maturation of the digestive and respiratory systems (Peña and Dumas 2009). TAL, on the other hand, transitioned from negative to isometric growth, indicating that it had reached sufficient size for effective locomotion and then maintained proportional growth with body length (Kupren et al. 2014; Saemi‐Komsari et al. 2018). This stabilization of tail development suggests that functional performance requirements were met early, a trend also reported in other cichlids (Saemi‐Komsari et al. 2018; Contreras‐Tapia et al. 2024b).

## 5 Conclusions

The early development of *Cryptoheros spilurus* larvae is characterized by a sequence of coordinated morphological, functional, and sensory transformations, reflecting the complex demands of transitioning through metamorphosis. Head development progresses rapidly, with the early appearance and spatial organization of key cranial structures such as the eyes, olfactory pits, cement glands, and oral cavity, accompanied by significant craniofacial remodeling. Buccal structures, including specialized dentition and taste buds, emerge in synchrony with the formation of an extensible upper lip, suggesting early adaptation toward benthic or scraping feeding modes. Associated changes in trunk morphology, notably the regionalization of pigmentation patterns and the flexion of the notochord, support the establishment of body axis functionality. Fin development occurs sequentially, with precocious pectoral fin maturation facilitating initial motility, while the caudal, dorsal, anal, and pelvic fins progressively develop to enable full locomotor capability. The transitory cement glands provide initial adhesion mechanisms, with their regression corresponding temporally to increased swimming skills. The emergence of superficial neuromasts and the deepening of the olfactory pit underscore the rapid onset of mechanosensory and chemosensory competence, essential for environmental interaction post‐hatching. Ontogenetic allometric analysis reveals shifts in morphological growth patterns during larval progression, with the consistent positive allometry of gape size (GS) aligning with the onset of active foraging and digestive demands.

This study suggests that the early developmental trajectory plays a fundamental role in the ecological success of the species. The integration of morphological and ultrastructural data reveals how these traits facilitate a critical transition from yolk dependence to active foraging, underscoring their adaptive significance in early survival. These findings highlight the importance of ontogenetic research in understanding cichlid diversification and suggest that similar developmental strategies may underlie broader patterns of adaptive radiation within Neotropical cichlids. While this study does not directly address phenotypic plasticity in response to ecological variation, it provides essential baseline data necessary for future studies exploring how developmental plasticity may contribute to ecological adaptation and diversification in *Cryptoheros spilurus* and related species. Future comparative studies within and between cichlid genera will clarify whether these traits represent a conserved developmental pathway or lineage‐specific adaptations, while also offering insights for conservation strategies targeting vulnerable early‐life stages.

## Ethics Statement

All experimental conformed to ethical standards for animal research as outlined in the Mexican Official Standard NOM‐062‐ZOO‐1999. Additionally, guidelines from The International Council for Laboratory Animal Science (ICLAS) were rigorously followed.

## Conflicts of Interest

The authors declare no conflicts of interest.

## Supporting information


**Table 1:** Summary of early larval development in *Cryptoheros spilurus* from hatching to 16 dph. The table includes the total length (TL) in mm and the main morphological and functional characteristics observed at each developmental stage.

## Data Availability

Derived data supporting the findings of this study are available from the corresponding author, on request.

## References

[ede70019-bib-0001] Albertson, R. C. 2003. “Genetic Basis of Adaptive Shape Differences in the Cichlid Head.” Journal of Heredity 94, no. 4: 291–301. 12920100 10.1093/jhered/esg071

[ede70019-bib-0002] Albertson, R. C. , and T. D. Kocher . 2006. “Genetic and Developmental Basis of Cichlid Trophic Diversity.” Heredity 97, no. 3: 211–221. 16835594 10.1038/sj.hdy.6800864

[ede70019-bib-0003] Alda, F. , W. B. Ludt , D. J. Elías , C. D. McMahan , and P. Chakrabarty . 2021. “Comparing Ultraconserved Elements and Exons for Phylogenomic Analyses of Middle American Cichlids: When Data Agree to Disagree.” Genome Biology and Evolution 13, no. 8: evab161. 34272856 10.1093/gbe/evab161 PMC8369075

[ede70019-bib-0004] Artigas‐Azas, J. M. 2007. “The Blue‐Eyed Cichlid, *Cryptoheros spilurus* (Günther, 1862).” Cichlid News Magazine 16, no. 1: 13–17.

[ede70019-bib-0005] Balon, E. K. 1999. “Alternative Ways to Become a Juvenile or a Definitive Phenotype (and on Some Persisting Linguistic Offenses).” Environmental Biology of Fishes 56, no. 1: 17–38.

[ede70019-bib-0006] Becker, E. A. , N. C. Bird , and J. F. Webb . 2016. “Post‐Embryonic Development of Canal and Superficial Neuromasts and the Generation of Two Cranial Lateral Line Phenotypes.” Journal of Morphology 277, no. 10: 1273–1291. 27519545 10.1002/jmor.20574

[ede70019-bib-0007] Beriotto, A. C. , P. G. Vissio , E. Gisbert , et al. 2023. “From Zero to Ossified: Larval Skeletal Ontogeny of the Neotropical Cichlid Fish *Cichlasoma dimerus* .” Journal of Morphology 284, no. 10: e21641. 37708507 10.1002/jmor.21641

[ede70019-bib-0008] Bird, N. C. , and J. F. Webb . 2014. “Heterochrony, Modularity, and the Functional Evolution of the Mechanosensory Lateral Line Canal System of Fishes.” EvoDevo 5: 21. 24959342 10.1186/2041-9139-5-21 PMC4066827

[ede70019-bib-0009] Boglione, C. , E. Gisbert , P. Gavaia , et al. 2013. “Skeletal Anomalies In Reared European Fish Larvae and Juveniles. Part 2: Main Typologies, Occurrences and Causative Factors.” Supplement, Reviews in Aquaculture 5: S121–S167.

[ede70019-bib-0010] Britz, R. , F. Kirschbaum , and A. Heyd . 2000. “Observations on the Structure of Larval Attachment Organs in Three Species of Gymnotiforms (Teleostei: Ostariophysi).” Acta Zoologica 81, no. 1: 57–67.

[ede70019-bib-0011] Buege, E. A. , P. C. Esselman , and S. J. Praskievicz . 2021. “Hydrogeomorphological Controls on Reach‐Scale Distributions of Cichlid Nest Sites in a Small Neotropical River.” Ecology of Freshwater Fish 30, no. 2: 244–255.

[ede70019-bib-0012] Burress, E. D. 2015. “Cichlid Fishes as Models of Ecological Diversification: Patterns, Mechanisms, and Consequences.” Hydrobiologia 748, no. 1: 7–27.

[ede70019-bib-0013] Cahu, C. , J. Zambonino Infante , and T. Takeuchi . 2003. “Nutritional Components Affecting Skeletal Development in Fish Larvae.” Aquaculture 227, no. 1–4: 245–258.

[ede70019-bib-0014] Cochran‐Biederman, J. L. , and K. O. Winemiller . 2010. “Relationships Among Habitat, Ecomorphology and Diets of Cichlids in the Bladen River, Belize.” Environmental Biology of Fishes 88: 143–152.

[ede70019-bib-0015] Coleman, R. M. 1991. “Measuring Parental Investment in Nonspherical Eggs.” Copeia 1991, no. 4: 1092–1098.

[ede70019-bib-0016] Conith, M. R. , A. J. Conith , and R. C. Albertson . 2019. “Evolution of a Soft‐Tissue Foraging Adaptation in African Cichlids: Roles for Novelty, Convergence, and Constraint.” Evolution 73, no. 10: 2072–2084. 31418824 10.1111/evo.13824

[ede70019-bib-0017] Contreras‐Tapia, R. A. , G. Garza‐Mouriño , M. E. Castellanos‐Páez , M. Castillo‐Rivera , and M. I. Benítez‐Díaz Mirón . 2024. “Ingestion Rate, Prey Selectivity, and Growth of Larval *Vieja zonata* (Teleostei: Cichlidae) Co‐Fed Rotifers With Cladocerans.” Aquaculture Nutrition 2024, no. 1: 6424063. 39555526 10.1155/2024/6424063 PMC11300068

[ede70019-bib-0018] Contreras‐Tapia, R. A. , M. I. Benítez‐Díaz Mirón , G. Garza Mouriño , and M. E. Castellanos‐Páez . 2024. “From Hatching to Juvenile: Larval Development of *Vieja fenestrata* (Teleostei: Cichlidae).” Journal of Fish Biology 105, no. 6: 1588–1602. 39126256 10.1111/jfb.15898 PMC11650922

[ede70019-bib-0019] Day, R. D. , D. P. German , and I. R. Tibbetts . 2011. “Why Can't Young Fish Eat Plants? Neither Digestive Enzymes nor Gut Development Preclude Herbivory in the Young of a Stomachless Marine Herbivorous Fish.” Comparative Biochemistry and Physiology Part B: Biochemistry and Molecular Biology 158, no. 1: 23–29. 10.1016/j.cbpb.2010.09.010 20884371

[ede70019-bib-0020] Finn, R. N. , ed. 2020. Fish Larval Physiology. CRC Press.

[ede70019-bib-0021] Flegler‐Balon, C. 1989. “Direct and Indirect Development in Fishes — Examples of Alternative Life‐History Styles.” In Alternative Life‐History Styles of Animals. Perspectives in Vertebrate Science, Vol 6. edited by M. N. Bruton . Springer.

[ede70019-bib-0022] Fujimura, K. , and N. Okada . 2007. “Development of the Embryo, Larva and Early Juvenile of Nile Tilapia *Oreochromis niloticus* (Pisces: Cichlidae). Developmental Staging System.” Development, Growth and Differentiation 49, no. 4: 301–324. 10.1111/j.1440-169X.2007.00926.x 17501907

[ede70019-bib-0023] Gisbert, E. 1999. “Early Development and Allometric Growth Patterns in Siberian Sturgeon and Their Ecological Significance.” Journal of Fish Biology 54, no. 4: 852–862.

[ede70019-bib-0024] Groppelli, S. , R. Pennati , C. Sotgia , and F. De Bernardi . 2003. “Cement Gland Apparatus of the Angelfish *Pterophyllum scalare* (Teleostei, Cichlidae): Functional Morphology in Comparison With Adhesive Organs of Other Chordata.” Italian Journal of Zoology 70, no. 2: 133–139.

[ede70019-bib-0025] Guma'a, S. A. 1978. “The Food and Feeding Habits of Young Perch, *Perca fluviatilis*, in Windermere.” Freshwater Biology 8: 177–187.

[ede70019-bib-0026] Hall, B. K. 2012. “Parallelism, Deep Homology, and Evo‐Devo.” Evolution and Development 14, no. 1: 29–33. 23016972 10.1111/j.1525-142X.2011.00520.x

[ede70019-bib-0027] Hopwood, N. 2007. “A History of Normal Plates, Tables and Stages in Vertebrate Embryology.” International Journal of Developmental Biology 51, no. 1: 1–26. 17183461 10.1387/ijdb.062189nh PMC1885287

[ede70019-bib-0028] Hu, Y. , and R. C. Albertson . 2017. “Baby Fish Working Out: An Epigenetic Source of Adaptive Variation in the Cichlid Jaw.” Proceedings of the Royal Society B: Biological Sciences 284, no. 1860: 20171018. 10.1098/rspb.2017.1018 PMC5563811 28768892

[ede70019-bib-0029] Huang, Y. F. , B. L. Song , T. H. Deng , Q. Wang , Q. Shen , and L. G. Liu . 2021. “Ontogenetic Development, Allometric Growth Patterns, and Daily Increment Validation of Larvae and Juvenile *Culter alburnus* .” Environmental Biology of Fishes 104: 1593–1610.

[ede70019-bib-0030] Hulsey, C. D. , M. E. Alfaro , J. Zheng , A. Meyer , and R. Holzman . 2019. “Pleiotropic Jaw Morphology Links the Evolution of Mechanical Modularity and Functional Feeding Convergence in Lake Malawi Cichlids.” Proceedings of the Royal Society B: Biological Sciences 286, no. 1897: 20182358. 10.1098/rspb.2018.2358 PMC6408893 30963830

[ede70019-bib-0031] Iwamatsu, T. 2004. “Stages of Normal Development in the Medaka *Oryzias latipes* .” Mechanisms of Development 121, no. 7–8: 605–618. 15210170 10.1016/j.mod.2004.03.012

[ede70019-bib-0032] Karachle, P. K. , and K. I. Stergiou . 2012. “Morphometrics and Allometry in Fishes.” Morphometrics 2: 65–86.

[ede70019-bib-0033] Kratochwil, C. F. , M. M. Sefton , and A. Meyer . 2015. “Embryonic and Larval Development in the Midas Cichlid Fish Species Flock (*Amphilophus* Spp.): A New Evo‐Devo Model for the Investigation of Adaptive Novelties and Species Differences.” BMC Developmental Biology 15: 12. 25887993 10.1186/s12861-015-0061-1 PMC4352272

[ede70019-bib-0034] Krogdahl, A. , A. Sundby , and A. M. Bakke . 2011. “Gut Secretion and Digestion.” In Encyclopedia of Fish Physiology: From Genome to Environment, edited by A. Farrell . Academic Press.

[ede70019-bib-0035] Kupren, K. , M. Prusińska , D. Żarski , S. Krejszeff , and D. Kucharczyk . 2014. “Early Development and Allometric Growth in *Nannacara anomala* Regan, 1905 (Perciformes: Cichlidae) Under Laboratory Conditions.” Neotropical Ichthyology 12, no. 03: 659–665.

[ede70019-bib-0036] Mabee, P. M. , D. S. Cua , S. B. Barlow , and J. V. Helvik . 1998. “Morphology of the Hatching Glands in *Betta splendens* (Teleostei: Perciformes).” Copeia 1998, no. 4: 1021–1026.

[ede70019-bib-0037] Majoris, J. E. , M. A. Foretich , Y. Hu , et al. 2021. “An Integrative Investigation of Sensory Organ Development and Orientation Behavior Throughout the Larval Phase of a Coral Reef Fish.” Scientific Reports 11, no. 1: 12377. 34117298 10.1038/s41598-021-91640-2 PMC8196062

[ede70019-bib-0038] Marconi, A. , C. Z. Yang , S. McKay , and M. E. Santos . 2023. “Morphological and Temporal Variation in Early Embryogenesis Contributes to Species Divergence in Malawi Cichlid Fishes.” Evolution & Development 25, no. 2: 170–193. 36748313 10.1111/ede.12429 PMC10909517

[ede70019-bib-0039] Maurakis, G. E. , and E. G. Maurakis . 2017. “Microstructure of Attachment Mechanisms of Newly Hatched Larvae of Four Cyprinid Species With Comments on Terminology.” Virginia Journal of Science 68, no. 3: 5.

[ede70019-bib-0040] Meijide, F. J. , and G. A. Guerrero . 2000. “Embryonic and Larval Development of a Substrate‐Brooding Cichlid *Cichlasoma dimerus* (Heckel, 1840) under Laboratory Conditions.” Journal of Zoology 252, no. 4: 481–493.

[ede70019-bib-0041] Miller, B. S. , and A. W. Kendall . 2009. “Chapter 2. Development of Eggs and Larvae.” In Early Life History of Marine Fishes. University of California Press.

[ede70019-bib-0042] Molina‐Arias, A. 2011. “Larval Development of *Hypsophrys nicaraguensis* (Pisces: Cichlidae) Under Laboratory Conditions.” Revista de Biologia Tropical 59, no. 4: 1679–1684. 22208084 10.15517/rbt.v59i4.3430

[ede70019-bib-0043] Nelson, H. M. , G. C. Coffing , S. Chilson , et al. 2019. “Structure, Development, and Functional Morphology of the Cement Gland of the Giant Danio, *Devario malabaricus* .” Developmental Dynamics 248, no. 11: 1155–1174. 31310039 10.1002/dvdy.88

[ede70019-bib-0044] Osse, J. W. M. , and J. G. M. Van den Boogaart . 1995. “Fish Larvae, Development, Allometric Growth, and the Aquatic Environment.” ICES Marine Science Symposia 201: 21–34.

[ede70019-bib-0045] Osse, J. W. M. , and J. G. M. Van den Boogaart . 2004. “Allometric Growth in Fish Larvae: Timing and Function.” Development of Form and Function in Fishes and the Question of Larval Adaptation 201: 167–194.

[ede70019-bib-0046] Osse, J. W. M. , J. G. M. Van den Boogaart , G. M. J. Van Snik , and L. Van der Sluys . 1997. “Priorities During Early Growth of Fish Larvae.” Aquaculture 155, no. 1–4: 249–258.

[ede70019-bib-0047] Otsuka, M. , S. Sugita , D. Shimizu , M. Aoyama , and M. Matsuda . 2023. “Radial Polarity in the First Cranial Neuromast of Selected Teleost Fishes.” Journal of Morphology 284, no. 11: e21654. 37856275 10.1002/jmor.21654

[ede70019-bib-0048] Parichy, D. M. , M. R. Elizondo , M. G. Mills , T. N. Gordon , and R. E. Engeszer . 2009. “Normal Table of Postembryonic Zebrafish Development: Staging by Externally Visible Anatomy of the Living Fish.” Developmental Dynamics 238, no. 12: 2975–3015. 19891001 10.1002/dvdy.22113 PMC3030279

[ede70019-bib-0049] Peña, R. , and S. Dumas . 2009. “Development and Allometric Growth Patterns During Early Larval Stages of the Spotted Sand Bass *Paralabrax maculatofasciatus* (Percoidei: Serranidae).” Scientia Marina 73, no. S1: 183–189.

[ede70019-bib-0050] Pottin, K. , C. Hyacinthe , and S. Rétaux . 2010. “Conservation, Development, and Function of a Cement Gland‐Like Structure in the Fish *Astyanax mexicanus* .” Proceedings of the National Academy of Sciences 107, no. 40: 17256–17261. 10.1073/pnas.1005035107 PMC2951400 20855623

[ede70019-bib-0051] Powder, K. E. , and R. C. Albertson . 2016. “Cichlid Fishes as a Model to Understand Normal and Clinical Craniofacial Variation.” Developmental Biology 415, no. 2: 338–346. 26719128 10.1016/j.ydbio.2015.12.018 PMC4914429

[ede70019-bib-0052] Powder, K. E. , K. Milch , G. Asselin , and R. C. Albertson . 2015. “Constraint and Diversification of Developmental Trajectories in Cichlid Facial Morphologies.” EvoDevo 6: 25. 26225206 10.1186/s13227-015-0020-8 PMC4518560

[ede70019-bib-0053] Prazdnikov, D. V. 2024. “The Role of Heterochrony in the Evolution of Pigment Patterns in Neotropical Freshwater Fishes: Experimental Evidence From Cichlidae and Poeciliidae.” Paleontological Journal 58, no. 12: 1466–1473.

[ede70019-bib-0054] Prazdnikov, D. V. , and F. N. Shkil . 2023. “The Role of Thyroid Hormones in the Development of Coloration of Two Species of Neotropical Cichlids.” Journal of Experimental Biology 226, no. 14: jeb245710. 37357638 10.1242/jeb.245710

[ede70019-bib-0055] Říčan, O. , L. Piálek , K. Dragová , and J. Novák . 2016. “Diversity and Evolution of the Middle American Cichlid Fishes (Teleostei: Cichlidae) With Revised Classification.” Vertebrate Zoology 66: 1–102.

[ede70019-bib-0056] Rønnestad, I. , M. Yúfera , B. Ueberschär , L. Ribeiro , Ø. Sæle , and C. Boglione . 2013. “Feeding Behaviour and Digestive Physiology in Larval Fish: Current Knowledge, and Gaps and Bottlenecks in Research.” Reviews in Aquaculture 5: S59–S98.

[ede70019-bib-0057] Saemi‐Komsari, M. , H. Mousavi‐Sabet , C. F. Kratochwil , M. Sattari , S. Eagderi , and A. Meyer . 2018. “Early Developmental and Allometric Patterns in the Electric Yellow Cichlid *Labidochromis caeruleus* .” Journal of Fish Biology 92, no. 6: 1888–1901. 29624691 10.1111/jfb.13627

[ede70019-bib-0058] Schmitz, A. , H. Bleckmann , and J. Mogdans . 2008. “Organization of the Superficial Neuromast System in Goldfish, *Carassius auratus* .” Journal of Morphology 269, no. 6: 751–761. 18431809 10.1002/jmor.10621

[ede70019-bib-0059] Schneider, R. F. , J. M. Woltering , D. Adriaens , and O. Roth . 2023. “A Comparative Analysis of the Ontogeny of Syngnathids (Pipefishes and Seahorses) Reveals How Heterochrony Contributed to Their Diversification.” Developmental Dynamics 252, no. 5: 553–588. 36351887 10.1002/dvdy.551

[ede70019-bib-0060] Sfakianakis, D. G. , E. Renieri , M. Kentouri , and A. M. Tsatsakis . 2015. “Effect of Heavy Metals on Fish Larvae Deformities: A Review.” Environmental Research 137: 246–255. 25594493 10.1016/j.envres.2014.12.014

[ede70019-bib-0061] Sibbing, F. A. , and F. Witte . 2005. “Adaptations to Feeding in Herbivorous Fish (Cyprinidae and Cichlidae).” In Periphyton: Ecology, Exploitation and Management, edited by M. E. Azim , M. C. J. Verdegem , A. A. van Dam , and M. C. M. Berveridge . CABI Publishing.

[ede70019-bib-0062] Van Snik, G. M. J. , J. G. M. Van Den Boogaart , and J. W. M. Osse . 1997. “Larval Growth Patterns in *Cyprinus carpio* and *Clarias gariepinus* With Attention to the Finfold.” Journal of Fish Biology 50, no. 6: 1339–1352.

[ede70019-bib-0063] Valtierra‐Vega, M. T. , and J. J. Schmitter‐Soto . 2000. “Feeding Habits of Cichlid Species (Perciformes: Cichlidae) in Caobas Lake, Quintana Roo, Mexico.” Revista de Biologia Tropical 48, no. 2–3: 503–508. 11354957

[ede70019-bib-0064] Webb, J. F. 2023. “Structural and Functional Evolution of the Mechanosensory Lateral Line System of Fishes.” Journal of the Acoustical Society of America 154, no. 6: 3526–3542. 38171014 10.1121/10.0022565 PMC10908562

[ede70019-bib-0065] Woltering, J. M. , M. Holzem , R. F. Schneider , V. Nanos , and A. Meyer . 2018. “The Skeletal Ontogeny of *Astatotilapia burtoni*–A Direct‐Developing Model System for the Evolution and Development of the Teleost Body Plan.” BMC Developmental Biology 18: 8. 29614958 10.1186/s12861-018-0166-4 PMC5883283

[ede70019-bib-0066] Yang, G. , Z. Wang , J. He , W. Li , and D. Yuan . 2020. “Allometric Growth Patterns of Fine Scale Fish Larvae and Its Ecological Significance.” Journal of Physics: Conference Series 1575, no. 1: 012157.

[ede70019-bib-0067] Yúfera, M. , and M. J. Darias . 2007. “The Onset of Exogenous Feeding in Marine Fish Larvae.” Aquaculture 268, no. 1–4: 53–63.

