# Supplementary material for: The Early Development of the Blue‐Eye Cichlid, Cryptoheros spilurus (Cichliformes: Cichlidae)
Source: Evol Dev. 2025 Oct 4;27(4):e70019. doi: 10.1111/ede.70019 (PMC12495903; doi:10.1111/ede.70019)
Supplement: Supplementary file 1 — Table 1: Summary of early larval development in Cryptoheros spilurus from hatching to 16 dph. The table includes the total length (TL) in mm and the main morphological and functional characteristics observed at each developmental stage. [file EDE-27-e70019-s001.docx]

**Table 1.** Summary of early larval development in *Cryptoheros spilurus* from hatching to 16 dph. The table includes the total length (TL) in mm and the main morphological and functional characteristics observed at each developmental stage.

| **Day Post Hatch (dph)** | **TL (mm)** | **Main Developmental Characteristics** |
| --- | --- | --- |
| 0 (Hatching) | 4.739 ± 0.27 | - Head ventrally flexed; mouth closed. |
|  |  | - Optic primordia and lenses visible (unpigmented). |
|  |  | - Otic vesicles, olfactory pits, and cement glands present. |
|  |  | - Continuous finfold around trunk and tail. |
|  |  | - Pectoral fin buds begin to form. |
|  |  | - Straight notochord; strong vascularization in finfold. |
|  |  | - Terminal gut and anal opening visible. |
|  |  | - Olfactory pit ~10 µm deep. |
|  |  | - Ventral melanophore stripe and large dendritic melanophores over yolk. |
| 1 | 5.214 ± 0.20 | - Head straightens; eye pigmentation begins. |
|  |  | - External gill openings visible. |
|  |  | - Pectoral fins continue development. |
|  |  | - Notochord begins flexion. |
|  |  | - Urogenital differentiation not yet evident. |
| 2 | 5.756 ± 0.21 | - Iridophores appear in the iris. |
|  |  | - Xanthophores emerge cranially and dorsally. |
|  |  | - Urinary bladder becomes distinguishable. |
|  |  | - Posterior finfold expands; caudal lobe begins forming. |
|  |  | - Vascularization in finfold decreases. |
| 3 | 5.856 ± 0.26 | - Mouth reaches terminal position; open and functional. |
|  |  | - Gill function observed; buccal ventilation begins. |
|  |  | - Urogenital pore differentiated from anus. |
|  |  | - Neuromasts (SNs) appear (SO, SUN, SM regions). |
|  |  | - Caudal fin: dorsal lobe protrudes, first lepidotrichia visible. |
|  |  | - Dorsal fin bulging observed. |
|  |  | - Eye pigmentation complete; pigmentation increases in trunk and pharyngeal region. |
| 4 | 6.194 ± 0.23 | - Branchiostegal rays emerge. |
|  |  | - Caudal ray segmentation visible. |
|  |  | - Head elongates and widens. |
|  |  | - Cement glands begin medial migration. |
|  |  | - Cranial melanophores and ventral pigmentation increase. |
| 5 | 6.365 ± 0.13 | - Notochord flexion completed; dorsal and ventral caudal lobes symmetric. |
|  |  | - Anal fin bulging visible. |
|  |  | - Free swimming. |
|  |  | - Cement glands medially aligned. |
|  |  | - Ventral stripe extends to tail; mandibular melanophores appear. |
| 6 | 6.619 ± 0.25 | - Mouth elongation evident; G-type teeth observed with mucus secretion. |
|  |  | - Dorsal fin rays first visible. |
|  |  | - Pelvic fin buds appear. |
|  |  | - Olfactory pit deepens (92 µm). |
|  |  | - Lateral and ventral pigmentation gains dashed appearance. |
| 7 | 6.949 ± 0.23 | - Yolk fully resorbed.  - Head elongation and widening continue. |
|  |  | - Pectoral fins fully formed with segmented rays. |
|  |  | - Cement glands flattened and nearly regressed. |
|  |  | - Basicaudal melanophores appear. |
| 8–9 | 7.494 ± 0.43 - 7.889 ± 0.26 | - Anal fin: lepidotrichia appear (9 dph). |
|  |  | - Continued fin ray segmentation. |
|  |  | - General pigmentation and fin structures mature. |
| 10–13 | 8.017 ± 0.31 - 10.272 ± 0.39 | - Steady growth and refinement of fins. |
|  |  | - Pelvic-fin rays visible. |
|  |  | - Continued body elongation and pigmentation patterning. |
| 14 | 10.974 ± 0.46 | - Dorsal and anal fins fully developed with unbranched and hard rays. |
|  |  | - Caudal fin reaches final shape. |
| 15–16 | 12.029 ± 0.50 - 13.168 ± 0.55 | - Pelvic fins fully developed with segmented rays. |
|  |  | - Pigmentation assumes vertical stripe pattern. |
|  |  | - Morphological structures as in the juvenile stage. |
